# A fast and lightweight tool for partially coherent beamline simulations in fourth-generation storage rings based on coherent mode decomposition

**DOI:** 10.1107/S1600577522008736

**Published:** 2022-10-18

**Authors:** Manuel Sanchez del Rio, Rafael Celestre, Juan Reyes-Herrera, Philipp Brumund, Marco Cammarata

**Affiliations:** a ESRF – The European Synchrotron, 71 Avenue des Martyrs, 38000 Grenoble, France; University of Tokyo, Japan

**Keywords:** partial coherence, undulator radiation, wave optics, beamline simulation, *OASYS*

## Abstract

A new algorithm to perform coherent mode decomposition of undulator radiation is proposed. It is based on separating the horizontal and vertical directions, reducing the problem by working with one-dimension wavefronts. The validity conditions of this approximation are discussed. Simulations require low computer resources and run interactively on a laptop. The focusing with lenses of the radiation emitted by an undulator in a fourth-generation storage ring (EBS-ESRF) is studied. Results are compared against multiple optics packages implementing a variety of methods for dealing with partial coherence: full two-dimension coherent mode decomposition, Monte Carlo combination of wavefronts from electrons entering the undulator with different initial conditions, and hybrid ray-tracing correcting geometrical optics with wave optics.

## Abstract 2

A new fast and lightweight software tool *WOFRY1D* for coherent mode decomposition of undulator emission is described. It is used to simulate focusing partially coherent beams with X-ray lenses. Results are analyzed and compared with other software packages (*COMSYL*, *SRW* and *ShadowOui*).

## 1. Introduction

The migration to fourth-generation storage rings has significantly improved the brilliance and coherence of X-ray synchrotron sources. The transverse coherent fraction of the new sources is increased by at least one order of magnitude with respect to the third-generation sources (typically from 10^−3^ to 10^−2^ at 10 keV). This has a beneficial impact^1^ for many applications requiring coherent beams, such as X-ray photon correlation spectroscopy, coherent diffraction imaging, propagation-based phase-contrast imaging, and ptychography (Paganin, 2006 ▸). However, the diffraction effects produced by the interaction of the beam with the edges and distortions of the optical elements strongly affect the quality of the beam. Diffraction patterns show higher visibility due to the increased coherent fraction in new sources, and its accurate modelling is fundamental for the design and optimization of beamlines. The physical models for the limiting cases of full incoherence (usually simulated by geometrical ray-tracing) or by propagating a single wavefront (valid for fully coherent radiation) are not sufficient for a complete understanding of the beam transport (Sanchez del Rio *et al.*, 2019 ▸). The coherent fraction of the radiation emitted by new generation storage rings, although much improved with respect to previous generations, is still of the order of a few percent at hard X-rays, which means that it is mandatory to account for partial coherence. In recent years, several modelling approaches have been demonstrated to work for beamlines using undulator radiation. Starting from incoherent beams, Shi *et al.* (2014 ▸) proposed some correction algorithms to include diffraction effects that occur with coherent radiation. More accurate methodologies exploit the well known propagation of coherent wavefronts. The partial coherence is treated by propagating a set of wavefronts that all together describe the undulator radiation. Two approaches are possible. One consists of calculating the wavefronts emitted by electrons entering the undulator with different initial conditions, sampled by Monte Carlo from the electron beam emittance (multi-electron in *SRW*) (Chubar *et al.*, 2011 ▸). A second method, the coherent mode decomposition (CMD), assigns these wavefronts to the coherent modes, which are the eigenfunctions of the cross-spectral density (CSD), and can be numerically calculated for the undulator source (Glass & Sanchez del Rio, 2017 ▸). A full treatment of CMD with two-dimensional (2D) wavefronts was implemented a few years ago in the *COMSYL* package (Glass, 2017*a* ▸). Both methods require the use of high-performance computer (HPC) resources that are not always at hand. The problem in CMD is to manipulate and diagonalize a huge stack representing the CSD with enough precision, which is a four-dimensional (4D) entity when using 2D wavefronts. After the release of *COMSYL*, different techniques have been proposed to deal with the magnitude of the problem. The single-value-decomposition method presents some advantages when used for the diagonalization of the CSD (Xu *et al.*, 2022 ▸). When the wavefronts are highly convergent or divergent, sufficient sampling of the electric field phase requires a very fine grid. In these cases, the sampling is dictated by a quadratic phase. A method developed by Li & Chubar (2022 ▸) consists of subtracting the quadratic phase which is analytically propagated, thus reducing the computational effort to limits acceptable by an average laptop.

In this paper, we propose a new method for dealing with partial coherence of undulator beams. The key point is to reduce the dimensionality of the problem to deal with one-dimensional (1D) wavefront cuts (*i.e.* separating horizontal and vertical directions).

We demonstrate that whenever this 1D approximation can be used, like in many cases of practical interest, the results are comparable with the other 2D methods but require much fewer resources, thus allowing simulations using a standard laptop.

The new code, referred to here as *WOFRY1D*, is benchmarked against other existing codes that are available in the *OASYS* simulation ecosystem (Rebuffi & Sanchez del Rio, 2017 ▸). The optical system studied here derives from the project for the new EBSL1 beamline at the upgraded EBS-ESRF storage ring. We have compared results for different set-ups implementing two refractive systems (transfocators), plus a slit placed upstream of the transfocators. The beam properties simulated by four different transfocator configurations are studied in detail using four packages available in *OASYS*: (i) the novel 1D CMD, implemented in the code *WOFRY1D*, (ii) full CMD in 2D with *COMSYL* (Glass, 2017*a* ▸), (iii) *SRW-ME*: multi-electron simulations in *SRW* (Chubar & Elleaume, 1998 ▸), and (iv) *HYBRID* ray-tracing as described by Shi *et al.* (2014 ▸) and implemented in *ShadowOUI* (Rebuffi & Sanchez del Rio, 2016 ▸).

## 2. Methods for describing partial coherent beams from undulators in a storage ring

In this section, we summarize the basic theory underneath partially coherent emission from electrons in storage rings. We start by showing that a relativistic single electron emits fully coherent radiation when passing through an undulator magnetic field. We then move to the emission from relativistic electron bunches showing that an electron beam with non-negligible emittance will produce a partially coherent emission and that a higher coherent fraction is associated with a lower electron-beam emittance. Finally, we present the basic principles underlining the numeric calculations within the packages used.

### 2.1. Description of undulator emission

**Figure 1:**
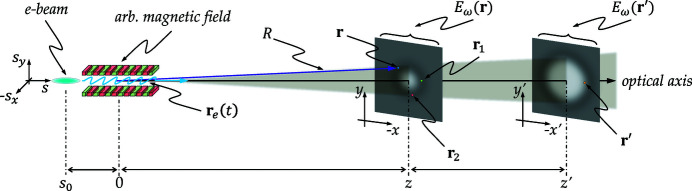
Spontaneous emission of relativistic electrons and propagation of wavefronts along the beamline.

We quickly remind that an ultrarelativistic charged particle following a curved trajectory [usually wiggly as produced by alternated magnetic fields in insertion devices (IDs)] emits radiation. This electric field can be calculated in the framework of classical electrodynamics [see, for example, equation (14.14) of Jackson (1999 ▸)]. In the frequency domain, the electric field at an observation point **r** = (*x*, *y*, *z*) can be written as

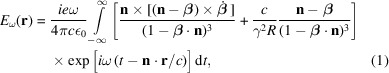

where the subscript ω indicates the photon frequency, *e* is the electron charge, *c* the velocity of light, ε_0_ the electric constant, γ ≃ 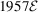 [GeV] is the Lorentz factor with 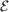 being the electron energy in practical units, **β** = 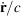 is the electron relative velocity and the dot denotes the time derivative. Also **n**(*t*) = **r** − **r**_e_(*t*)/|**r** − **r**_e_(*t*)| is the unit vector pointing from the particle to the observation point **r**; the electron trajectory is represented by **r**_e_(*t*), which is completely determined by the 3D distribution of the magnetic field inside the ID and the electron initial conditions prior to entering it. The origin of the vector **r** is usually at the centre of the insertion-device/straight-section. Figure 1 ▸ serves as a visual aid to equation (1) and its parameters.

Equation (1) describes a fully spatially coherent field and has been conceptualized for a single electron. A common abstraction that derives from it is the ‘filament beam’, where *N*_e_ electrons overlap in space following the same trajectory **r**_e_(*t*), which is useful to represent an idealized zero-emittance storage ring. In this case, a multiplicative factor *N*_e_ is applied to equation (1). Much like the single electron emission, the filament beam also radiates a fully transverse coherent wavefront.

Several codes are available in the synchrotron community to calculate the undulator emission characteristics in different cases. The codes *URGENT* (Walker & Diviacco, 1992 ▸) and *US* (Dejus & Luccio, 1994 ▸) compute undulator emission in the far-field for undulators with a sinusoidal magnetic field. The codes *SPECTRA* (Tanaka & Kitamura, 2001 ▸) and *SRW* (Chubar & Elleaume, 1998 ▸) are more generic as they calculate emission in the near- and far-field for any electron trajectory (with different initial conditions) and submitted to an arbitrary magnetic field.

### 2.2. Electron beam distribution in storage rings

At any position *s* in the storage ring, an electron can be described by five coordinates, 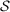 = (*x*_e_, 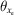 , *y*_e_, 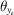 , 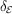 ), representing the phase space coordinates and a term 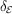 expressing the relative deviation of the electron energy from the main storage ring energy (also known as the energy spread). It follows that at any given *s* the many electrons in a bunch follow a 5D Gaussian distribution,

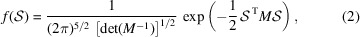

with *M* as the inverse of the generalized variance 5 × 5 matrix. A common assumption is that the variables are correlated only if they are in the same plane (*x* or *y*). In some particular points *s* where the covariance between spatial and angle terms is zero, only the diagonal terms in *M* are non-zero: ( 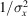 , 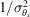 , 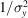 , 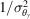 , 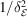 ). This is usually the case at the centre of the straight sections, where the undulators are often placed. We also assume that the electron bunch has a Gaussian distribution with σ_*z*_ along the longitudinal direction, as most particle beams do [*cf*. §8 in Wiedemann (2019 ▸)].

### 2.3. Emission from electron bunches

Having summarized the coherent emission from a single electron in Section 2.1 and how the electrons are statistically distributed in a bunch (Section 2.2), we now turn our attention to the emission from the electron bunch with finite emittance.

The total electric field emitted from all *N*_e_ electrons in a bunch circulating in a storage ring is given by 

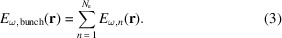

In terms of intensity,

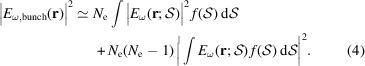

The electric field 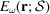 is the emission by a single electron with a trajectory defined by the undulator magnetic field and electron initial conditions 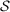 at the observation point **r** [see equation (1)]. The first term in equation (4) is a sum at **r** of the intensity of every electron emission weighted by its probability *f*, which describes temporally incoherent synchrotron radiation (SR). The second term stands for the enhancement of the intensity due to coherent superposition of the emission of the *N*_e_ electrons, modelling temporally coherent synchrotron radiation. It follows that *I*_bunch_ = *I*_iSR_ + *I*_cSR_. For emitted wavelengths λ shorter than the electron bunch length (σ_*z*_ > λ), the power associated with the term *I*_cSR_ vanishes quickly (Hirschmugl *et al.*, 1991 ▸; Wiedemann, 2019 ▸). Considering typical undulator radiation emission, *i.e.* X-ray energy ranges from a few hundred electronvolts to a few hundred keV, and typical electron bunch lengths in storage rings (σ_T_ > 30 ps), *I*_cSR_ can be neglected when considering standard monochromatization schemes in beamlines.^2^

Similarly, the mutual correlation of the electric field between two observation points **r**_1_ and **r**_2_ is

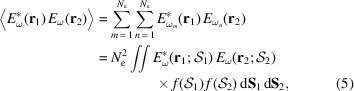

where the superscript asterisk ( 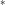 ) indicates the complex conjugate, the angular brackets 〈…〉 indicate the sum over the bunches, **r**_1_ = (*x*_1_, *y*_1_, *z*_1_) and **r**_2_ = (*x*_2_, *y*_2_, *z*_2_) (see Fig. 1 ▸). This equation is the cross-spectral density, that will be discussed later and rewritten in a more manageable form.

### 2.4. Multi-electron Monte Carlo (*SRW-ME*)

Synchrotron radiation emitted by undulators in storage rings is a fundamentally random process and should be treated probabilistically. The *SRW-ME* algorithm used to account for partial coherence implements equations (4) and (5) by individually calculating the synchrotron emission (electric field) of several electrons subjected to the initial conditions sampled from 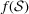 assuming these are uncorrelated and passing through an arbitrary magnetic field describing the X-ray source. Each resulting electric field from this Monte Carlo sampling is then propagated through the beamline until the observation point, where the contributions from different electrons are added in intensity (Chubar *et al.*, 2011 ▸). It is impractical (and unnecessary) to account for the emission of every single electron in a beam that very often has a current of a few hundred mA. Electrons are then divided into so-called macro-electrons (me), which is an abstraction that allows the emission of several individual electrons to be grouped into one ‘superparticle’ emitting a fully coherent wavefront but with resulting intensity given by the total intensity *I*_bunch_ divided by the number of macro-electrons 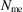 used in the simulation,

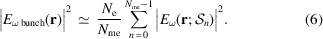

An advantage of the *SRW-ME* approach is that, since the electric fields of the macro-electrons propagate independently from each other, a convenient parallelization of the wavefront propagations among many processors is possible, requiring the use of HPC in most cases.

### 2.5. Coherent mode decomposition of undulator radiation

The cross-spectral density, generally represented as 

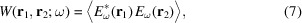

expresses the correlation of the emitted radiation between any two spatial points **r**_1_ and **r**_2_. It is the fundamental object that we will use to describe all partially coherent properties of the synchrotron beams. We justify first, in the context of the existing literature, the conditions of its usage for synchrotron light. Then we present the CMD and its practical implementation in 2D (with *COMSYL*) and the proposed 1D algorithm (with *WOFRY1D*).

#### 2.5.1. Validity of CSD usage for emission in storage rings

Geloni *et al.* (2008 ▸) show^3^ that, although synchrotron radiation emission (a random process obeying Gaussian statistics) is not intrinsically stationary nor homogeneous, the second-order coherence theory of scalar fields as presented by Mandel & Wolf (1995 ▸) can be applied when the following conditions are observed:

(1) Radiation frequency ω is ‘sufficiently high’.

(2) e-bunch time-length σ_T_ is ‘sufficiently large’ so that ωσ_T_ ≫ 1.

(3) Radiation bandwidth Δ_ω_ is not ‘too narrow’ (Δ_ω_ ≫ 1/σ_T_).

Excluding infra-red frequencies and below, condition (1) holds for soft and hard X-rays; condition (2) is satisfied by storage rings, where typically σ_T_ > 30 ps, but not at free-electron lasers, where σ_T_ < 0.1 ps due to micro-bunching effects; and, finally, condition (3) is generally met by standard monochromatization schemes. This set of conditions, related to the longitudinal electron-beam direction, ensures that synchrotron radiation emission is a quasi-stationary (or a wide-sense stationary) process.

In the (2D) transverse direction, following Geloni *et al.* (2008 ▸), the source is quasi-homogeneous when (i) the intensity slowly varies compared with the coherence length, and (ii) the complex degree of coherence depends only on Δ**r** = **r**_2_ − **r**_1_. The following additional conditions delimit the applicability of quasi-homogeneity:

(4) *N*_*x*_ ≫ 1 and/or *D*_*x*_ ≫ 1.

(5) *N*_*y*_ ≫ 1 and/or *D*_*y*_ ≫ 1, with

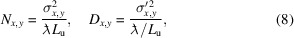

where σ_*x*,*y*_ and 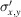 represent the electron beam transverse sizes and divergences, *L*_u_ is the undulator length (number of periods *N*_u_ times the magnetic period λ_u_) and 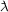 = λ/2π. If conditions (4) and (5) are met with ‘and’, we have a Gaussian quasi-homogeneous source. For third-generation synchrotron light sources, condition (4) is met with ‘and’ and condition (5) with ‘or’. Here the source is quasi-homogeneous and can be separated in H and V, as shown by Geloni *et al.* (2008 ▸) [equation (56)]. As the horizontal emittance reduces, as it is the case of fourth-generation light sources (for the cases studied here *N*_*x*_ = 13, *D*_*x*_ = 2.6, *N*_*y*_ = 0.4, *D*_*y*_ = 0.3), we slowly approach a region where quasi-homogeneity breaks down.

#### 2.5.2. Coherent modes, coherent fraction and coherent length

The CSD in equation (7) is used to define the spectral density^4^ as

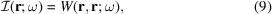

for the case where **r** = **r**_1_ = **r**_2_. We also define the normalized cross-spectral density function or spectral degree of coherence^5^, hereafter referred to as DoC, as

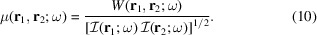

The modulus value of equation (10) is limited to 0 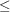 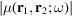 ≤ 1, where |μ| = 0 means total uncorrelation and |μ| = 1 denotes full correlation of the fluctuations at positions **r**_1_ and **r**_2_.

A well known result from coherence theory is the coherent mode representation of partially coherent fields in free space [see §4.7.1 in Mandel & Wolf (1995 ▸)]. It is possible to decompose the CSD into an infinite sum of orthonormal coherent modes,

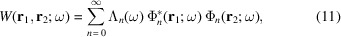

where Λ_*n*_ (eigenvalues) are the intensity weights and Φ_*n*_ are the coherent modes (eigenfunctions). Some important characteristics of this coherent mode decomposition are:

(1) The modes Φ_*n*_ are orthonormal in the integral sense.

(2) The decomposition maximizes the CSD making the truncation optimal,

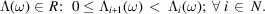

(3) The eigenvalues Λ_*n*_ are a measure of the intensity of the corresponding mode Φ_*n*_.

(4) We define the occupation η of the *i*th mode as its normalized intensity, 

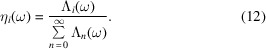

(5) Radiation is considered fully coherent if and only if there is only a single mode.

From these arguments, it is now natural to rigorously define coherent fraction (CF) as the occupation of the first coherent mode,

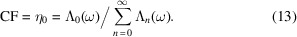

The transverse coherence length (CL) is related to the width of a cut of the modulus of the DoC. There is no unanimous accepted way of defining the CL, a parameter of practical importance for daily beamline operations. Quantitative values of CL are discussed in §5.1. Importantly, the blind application of the van Cittert–Zernike theorem may lead to errors, as discussed in §4.1 of Geloni *et al.* (2008 ▸), because this theorem was originally derived for incoherent sources.

The interest in the coherent mode decomposition method applied to the optical design of X-ray beamlines is manyfold: (i) the possibility of propagating a partially coherent beam along a beamline by just propagating coherent modes; (ii) the ability to compute the CSD and therefore all the related coherence parameters from the propagated modes; (iii) the use of the coherent fraction (a scalar parameter) as a well defined measure of how coherent is the beam at any position of the beamline; (iv) the numerical storage of the *N*_m_ modes that depend on two spatial variables is more economic than the storage of the CSD, a complex function of many variables; and (v) the infinite series converges smoothly to the CSD, therefore the truncation at a limited number of modes *N*_m_ always guarantees that it is the best possible reduced representation of the CSD and can be quantified.

#### 2.5.3. Coherent mode decomposition with *COMSYL*

*COMSYL* (*COherent Modes for SYnchrotron Light*) is a software package to perform the CMD of undulator radiation in a storage ring (Glass, 2017*a* ▸). A complete description of the code is given by Glass (2017*b* ▸) and here we summarize the principles underlying it. Applications of this software for beamline modelling at the ESRF-EBS are presented by Glass & Sanchez del Rio (2017 ▸) and Sanchez del Rio *et al.* (2019 ▸). *COMSYL* was used to study the specked structure of the CSD and the presence of X-ray coherence vortices and domain walls (Paganin & Sanchez del Rio, 2019 ▸).

Coherent mode decomposition consists of calculating Λ_*i*_ and Φ_*i*_ in equation (11). This operation can be seen as the ‘diagonalization’ of *W* where the eigenfunctions are the coherent modes (Φ_*i*_) and the eigenvalues their intensity weights (Λ_*i*_). These are the solution of the homogeneous Fredholm integral equation of the second kind, 

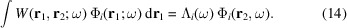

The eigenvalue in equation (14) can be written

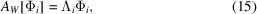

where we define the Hermitian operator for a generic function *g* as 

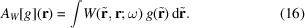

A first look at the CSD expression [see equation (5)] is enough to get an impression of how it is resource-intensive calculating and storing this 6D function. For synchrotron beams it is useful to decouple the longitudinal coordinate – along the optical axis in a beamline (see Fig. 1 ▸) – so that the CSD reduces to 4D, and wavefronts are 2D. We use *W*_2D_ notation for this CSD. Knowing the CSD at a given position *z*, that is, 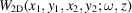 , it is possible to propagate it to another position *z*′ and also backpropagate to the source position.

Kim (1986 ▸) developed a propagation theory of synchrotron radiation using Wigner distribution. He introduced the ‘brightness convolution theorem’ stating that the source brightness due to a collection of electrons randomly distributed in their phase space is calculated by a convolution of the source brightness due to a reference electron with the electron distribution function. *COMSYL* applies Kim’s brightness convolution theorem in a plane (*s*_*x*_, *s*_*y*_) at *s*_0_ = −*L*_u_/2, which is where the electrons enter the undulator. This distance *s*_0_ is taken from the centre of the ID (origin of the optical axis) – see Fig. 1 ▸. We have [*cf*. equation (3.37) in Glass (2017*b* ▸)]

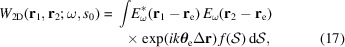

where **r**_e_ = (*x*_e_, *y*_e_), **θ**_e_ = 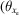 , 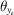 ) and Δ**r** = **r**_2_ − **r**_1_. The electric field *E*_ω_ is calculated using *SRW* in an arbitrary plane at a distance *z* and then backpropagated to *s*_0_ using the standard Fresnel free-space propagator (see Section S2 of the supporting information).

*COMSYL* starts with a matrix method that discretizes the cross-spectral density operator 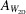 in a step function basis set [see equation (4.4) of Glass (2017*b* ▸)]. The discretization is followed by an iterative diagonalization using *SLEPc* (Hernandez *et al.*, 2005 ▸). The implementation uses parallel computation and requires the use of an HPC. The key point of *COMSYL* is to avoid storing the full representing matrix because it requires significant memory and computational resources. It scales essentially with 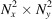 where *N*_*x*_ and *N*_*y*_ are the numbers of grid points in the *x* and *y* dimension, respectively. Typical sizes for *N*_*x*_ and *N*_*y*_ can easily reach a few hundred up to a few thousand. In the latter case, the memory requirements would reach several thousand terabytes. To reduce the memory requirements of the matrix method, *COMSYL* uses a two-step method that first performs a coherent mode decomposition for a zero divergence electron beam and based on this decomposition performs a second decomposition that takes the divergence into account. The memory requirement for our undulator applications is drastically reduced to about 4 × *N*_*x*_ × *N*_*y*_ × *N*_m_ where *N*_m_ is the number of requested coherent modes. This allows the calculation of higher harmonics or higher emittance rings where *N*_*x*_ × *N*_*y*_ ≫ *N*_m_.

The modes calculated with the just-described method can be propagated along the beamline and used to compute the spectral density and cross-spectral density at any point of the beamline. These modes can be conveniently propagated downstream of a beamline with *SRW* or *WOFRY* using the *OASYS* environment (see §3). However, due to modifications by optical elements (cropping/truncation and/or absorption), the propagated wavefronts generally lose their orthonormality. Thus, for computing the CF at a given point of the beamline, it is necessary to perform a new CMD with the propagated CSD. It is possible to apply the very same method used to compute the initial coherent modes, but *COMSYL* implements the action of the integral operator [equation (14)] directly to the coherent modes, which is much more economic in terms of memory usage.

#### 2.5.4. Coherent mode decomposition with separate horizontal and vertical directions (*WOFRY1D*)

Resuming the discussion in §2.5.1, we now assume to be in a quasi-homogeneous regime. This allows us to decompose the CSD as a product of horizontal and vertical cross-spectral densities,

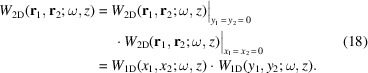

The CSD for the horizontal (*x*) direction is

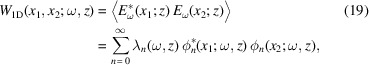

and similarly for the vertical direction (replacing *x* by *y*), with λ_*n*_ the eigenvalues (scalars) and ϕ_*n*_ the 1D eigenfunctions. The CSD described in equation (19) is now a 2D function. This dimension reduction is very welcome as it becomes very easy to store, manipulate and diagonalize the CSD using common tools (*e.g.* the Python numpy and SciPy libraries). Much like that presented in §2.5.3 for the *COMSYL* software, we calculate the 1D undulator emission at an arbitrary plane located at *z* from the origin using *pySRU* (Thery *et al.*, 2016 ▸), and backpropagate this field to the centre of the undulator using *WOFRY*’s zoom propagator (see Section S3 of the online supporting information). *W*_1D_ is then obtained using Kim’s brightness convolution theorem at *z* = 0, which is effectively constructed using equation (3.51) of Glass (2017*b* ▸).

This recipe is implemented in the *WOFRY* wavefront propagation add-on in *OASYS*. For a typical beamline, two calculations are done: one for the horizontal direction and another for the vertical. These two results can be combined to obtain the CSD 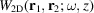 = 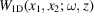 × 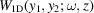 , implying numerical tensor operations. Similarly, the 2D spectral density is retrieved as 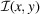 = 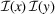 . This will be extensively used in later sections. Note that if the intensity is stored in arrays, the product is indeed an outer product. Finally, this decomposition gives rise to two values of coherence fraction: CF_*x*_ for the horizontal direction and CF_*y*_ for the vertical direction. The 2D CF can be retrieved as CF = CF_*x*_ × CF_*y*_. Like in *COMSYL*, the ϕ_*n*_ eigenfunctions can be propagated to any position of the beamline like standard wavefronts. The propagated *W*_1D_ can, again, be decomposed in coherent modes to obtain the CF after propagation.

The factorization in equation (18) has been discussed by Geloni *et al.* (2008 ▸) [see their §3.1, equation (56)] where it is stated that this rearranging of equation (7) is only valid for quasi-homogeneous sources (see §2.5.1). In the context of the Wigner distribution, this separation in horizontal and vertical components has been discussed by various authors and is believed to work well for undulator radiation with photon energies near the resonance of odd harmonics (Bazarov, 2012 ▸; Tanaka, 2014 ▸; Nash *et al.*, 2021 ▸). The Cartesian factorization in equation (18) is supposed to work well when the electron beam parameters in the horizontal are different from the vertical ones. The Cartesian separation does not work for rotationally symmetric sources (round beams). However, the Wigner function in this case (and therefore the CSD, being related by a Fourier transform) can be treated as a 1D problem, as suggested by Agarwal & Simon (2000 ▸) and developed by Gasbarro & Bazarov (2014 ▸). It would be interesting for future fourth-generation sources that will create round beams to develop a CMD tool using polar coordinates, also including wave propagators like those discussed by Li & Jacobsen (2015 ▸).

### 2.6. Hybrid ray-tracing (*HYBRID*)

Simulations methods using ray-tracing are simpler and faster than those using wave-optics, and they offer useful insight when studying and designing a synchrotron beamline (Sanchez del Rio *et al.*, 2019 ▸). However, pure ray-tracing methods (based on geometrical optics) are not adequate when analyzing optical systems dealing with (partially or fully) coherent beams subjected to strong diffraction effects (*e.g.* beam clipping by either physical acceptance of an optical element or by slits and optical errors). It is possible to add diffraction effects to the geometric methods, by convolution of the beam divergence profiles calculated by ray-tracing with those resulting from the diffraction integrals, then proceeding with classical ray-tracing methods. This hybrid concept (Shi *et al.*, 2014 ▸) is implemented as an extension to the ray-tracing code *SHADOW* (Sanchez del Rio *et al.*, 2011 ▸) available in the *ShadowOUI* (Rebuffi & Sanchez del Rio, 2016 ▸) add-on in *OASYS*. Since its release, this *HYBRID* ray-tracing implementation has been successfully used as an efficient and fast tool to design beamlines also including coherence effects (Shi *et al.*, 2017 ▸; Rebuffi & Shi, 2020 ▸; Lordano, 2022 ▸).

## 3. Propagation of wavefronts along the beamline

In terms of design using physical optics, an X-ray beamline is composed of two main elements: drift spaces and optical elements. The first category is handled with diffraction integrals, and a brief explanation of the wavefront propagators used by the software in Section 2 is presented here. Optical elements can usually be represented by transmission elements and their treatment is also covered in this section.

### 3.1. Drift spaces

Under the scalar theory, a generic wavefront obeying the wave equation and completely described at a position *z*, that is, *E*_ω_(**r**), known for all the *xy* plane, will propagate (evolve) between two parallel planes separated by a distance *L* = *z*′ − *z* as 

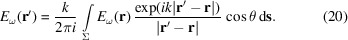

Equation (20) is the first Rayleigh–Sommerfeld diffraction equation (Huygens–Fresnel principle) and is valid for the case where |**r**′ − **r**| ≫ λ, with λ = 2π/*k*. We define a normal vector parallel to the optical axis (*z*-axis) **ℓ** so that θ is the angle between −**ℓ** and the vector **r**′ − **r**; Σ is the *xy* plane in *z* where the integration takes place with 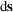 = 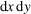 (see Fig. 1 ▸). Further simplification to equation (20) can be done using the paraxial approximation. In this case, it is assumed that 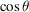 ≃ 1; and that the term |**r**′ − **r**| = [(*x*′ − *x*)^2^ + (*y*′ − *y*)^2^ + *L* ^2^]^1/2^ can be expanded in a Taylor series with *L* ^2^ ≫ (*x*′ − *x*)^2^ and *L* ^2^ ≫ (*y*′ − *y*)^2^. Retaining the quadratic term in the exponential function, but dropping it for the denominator,

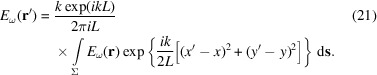

This approximation is known as the Fresnel diffraction integral and strategies for its numerical calculation are plenty (Kelly, 2014 ▸; Goodman, 2017 ▸); see also Rees (1987 ▸), Stern & Javidi (2004 ▸) and Zhang *et al.* (2020 ▸) for other practical considerations.

The different wave-optics packages in use have different implementations of these propagators. The selection of the propagator and its parametrization (sampling, padding, interpolation, *etc*.) is made depending on the particular characteristics of the optical element and propagation distances. *SRW* uses four different propagators, summarized in Section S2 of the supporting information. *WOFRY1D* basically uses two different propagators (see Section S3).

### 3.2. Optical elements

Optical elements will interact with the wavefront by manipulating its amplitude and phase. A very wide range of optical elements can be represented by the complex transmission element,

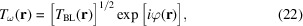

which is applied to the electric field *E*_ω_(**r**) as a multiplicative factor (Cloetens *et al.*, 1996 ▸). This thin-element approximation can be expanded to represent thick optical elements by applying the multi-slicing method, which represents an optical element as intercalation of several thin elements (slices) and free-space propagation between them (Paganin, 2006 ▸; Li *et al.*, 2017 ▸; Munro, 2019 ▸) (see Section S4 of the supporting information).

A generic aperture (slit, pin-hole and beam-stop) is represented by a binary opaque object: it masks part of the wavefront,

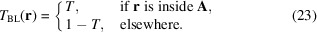

When the element is a slit, then *T* = 1. If it is a beamstop, then *T* = 0. **A** is the region defined by the object boundary.

Absorption filters, test patterns, X-ray refractive lenses, refractive correctors (free-form refractive optics), zone plates and transmission gratings are usually well represented by this thin-element approximation in projection approximation with 

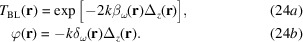

Δ_*z*_ is the projected thickness of an optical element along the *z*-axis. We define the index of refraction as *n*_ω_ = 1 − δ_ω_ + *i*β_ω_. Optical errors can be simulated in a similar way (Laundy *et al.*, 2014 ▸; Celestre *et al.*, 2020 ▸; Sanchez del Rio *et al.*, 2020 ▸).

Refractive systems, like the lenses used in this work, are calculated using the thin-object approximation with the lens described by its profile and refraction index (material). Usually, a single lens has a parabolic interface *z* = *x* ^2^/(4*R*) with *R* the radius at the apex. A lens has usually two parabolic interfaces (front and back) separated by a lens thickness *d*_L_. The interfaces are flat outside the lens aperture *A*. Therefore, the lens thickness profile is

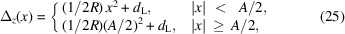

used to compute the complex transmission with equation (22).

Reflective optics can also be reasonably well approximated by transmission elements with more complex calculations, *e.g.* stationary-phase methods [application of equation (20) over the mirror surface], geometric ray-tracing calculation of the optical path difference (Canestrari *et al.*, 2014 ▸) or even multi-slicing (Li *et al.*, 2017 ▸). Alternatively, 1D wavefronts can be easily propagated in grazing-incidence mirrors and gratings calculating directly the numeric integral in equation (20) (Raimondi & Spiga, 2015 ▸; Sanchez del Rio *et al.*, 2020 ▸).

## 4. Description of the optical system

The optical system under consideration is based on the future ‘EBSL1’ beamline at ESRF.^6^ This will be a long beamline (200 m) for applications exploiting the beam coherence. The source considered is an undulator with 138 periods of 18 mm (length close to 2.5 m). We analyze a focusing system with two transfocators, at 65 and 170 m from the source. They contain sets of 2D and 1D lenses that will permit modifying independently the focal lengths for the horizontal and vertical directions. The use of two transfocators allows great flexibility in beam transport (Vaughan *et al.*, 2011 ▸). The first one can be used to modify the divergence of the beam, even to collimate it, to guarantee a full illumination at the second transfocator.

**Figure 2:**
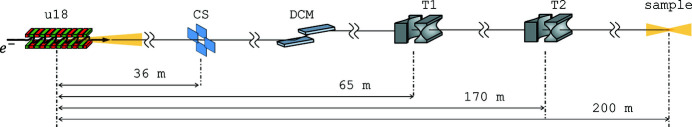
Schematic view of the beamline with the distances used in the simulations. The source is the undulator u18 set to *K* = 1.851 (7 keV at first harmonic). CS is the coherence slit that controls the coherent fraction. DCM is the double-crystal monochromator (not used in the calculations). T1 and T2 are the two transfocators, idealized in single parabolic lenses. The observation plane (sample) is 200 m from the source.

Each of the two transfocators in use (T1 and T2 in Fig. 2 ▸) are idealized as two crossed 1D Be lenses. For each plane (H and V), lens-1 and lens-2 have variable curvature radii *R*_1_ and *R*_2_ that match the focal distances *f*_1_ and *f*_2_ (*f* = *R*/2δ), with δ = 6.96 × 10^−6^ for Be at 7 keV. The focal lengths for the lenses are different for the horizontal and vertical directions to adapt to the beam characteristics. A slit (CS in Fig. 2 ▸) of aperture *a*_*x*_ in the horizontal and *a*_*y*_ in the vertical is placed upstream of the lens-1. We set the distances matching the requirements of the EBSL1 beamline (see Fig. 2 ▸), and we analyzed the system at a photon energy of 7 keV for different focal distances of lens-1 and lens-2.

We are interested in the beam properties (intensity distribution, size, flux) at the sample plane for four cases. The first case is selected to obtain a small spot (about 5 µm) and the second one a large spot (more than 30 µm). For these cases, the slits are selected to match CF_*x*_ = CF_*y*_ = 90% for a photon energy of 7 keV. The values are shown in Table 1 ▸. Cases 3 and 4 follow the same logic but the slits are opened to increase intensity at the expense of reducing coherence (CF_*x*_ = CF_*y*_ = 70%).

## 5. Results and discussion of multi-optics simulations

Calculations are done using the four different methodologies discussed previously, implemented in four different add-ons of the *OASYS* ecosystem.

### 5.1. Source characteristics and its propagation to the entrance slit

**Figure 3:**
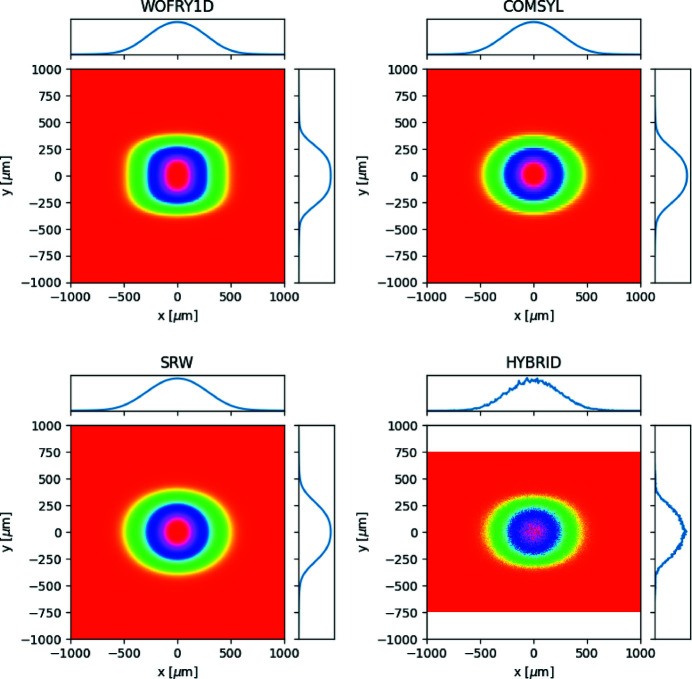
Spectral density at the slit plane (36 m from source) calculated by the four codes in use. The profiles correspond to the lines passing through (0, 0).

We first calculated the source and the illumination at the entrance slit plane (*z* = 36 m). The spectral density calculated with the different codes is shown in Fig. 3 ▸. The distributions calculated by the different methods are close with differences in full width at half-maximum (FWHM) of less than 10%.

**Figure 4:**
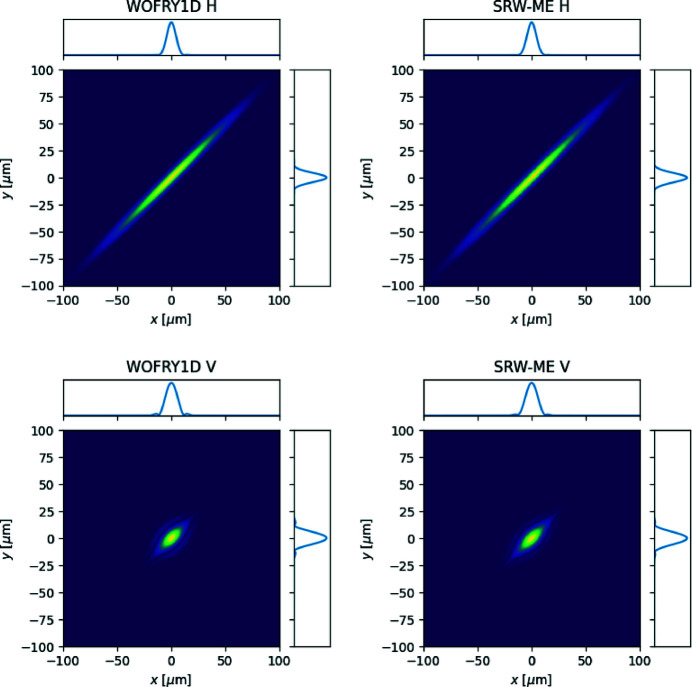
Cross-spectral density at the source plane (*z* = 0) calculated by *WOFRY1D* and *SRW-ME* for the horizontal (top row) and vertical (bottom row) directions. The profiles correspond to the lines passing through (0, 0).

The CSD can be calculated using wave optics codes. Figure 4 ▸ shows the horizontal and vertical *W*_1D_ at the source plane calculated with *SRW-ME* and *WOFRY1D*. Again, an excellent agreement is found, with similar FWHM of the profiles crossing (0, 0) [9 µm for both codes in the horizontal (H) and 12 µm in the vertical (V)].

**Figure 5:**
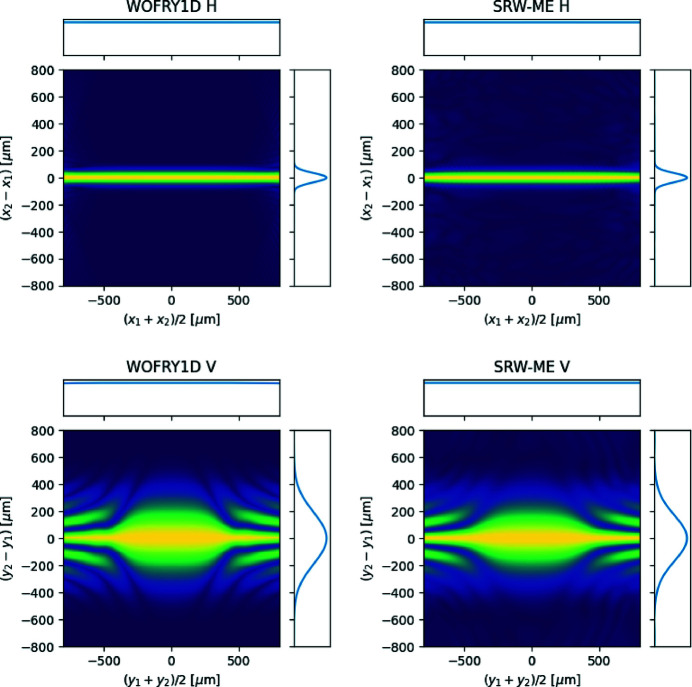
Modulus of the spectral degree of coherence at the slit plane (*z* = 36 m) calculated by *WOFRY1D* and *SRW-ME* for the horizontal (top row) and vertical (bottom row) directions. The profiles correspond to the lines passing through (0, 0).

Figure 5 ▸ shows the horizontal and vertical DoC at the slit plane expressed in the new coordinates ( 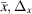 ) = [(*x*_1_ + *x*_2_)/2, *x*_2_ − *x*_1_] (for the horizontal, similarly with *y* for the vertical). The interest in using these coordinates is to redress the plot of the CSD that lies on a diagonal (as shown in Fig. 4 ▸) and obtain the ‘coherent length’ (CL) as a ‘width’ of the modulus of the DoC versus Δ_*x*_ at 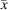 = 0. If using the FWHM, we obtain a horizontal CL of 76 µm with *WOFRY1D* and 80 µm with *SRW-ME* and a vertical CL of 444 µm with *WOFRY1D* and 402 µm with *SRW-ME*. A naive application of the van Cittert–Zernike theorem for a source with a Gaussian intensity profile permits the calculation of the coherence length (the FWHM of the Fourier transform of the source intensity profile) as CL = 0.88λ*z*/*S*, with *z* the source–observation plane distance, and *S* the source FWHM. In our case [*z* = 36 m, source FWHM 70.6 µm (H) and 15.0 µm (V), and λ = 1.77 × 10^−10^ m] it gives CL = 79 µm (H) and CL = 374 µm (V). The (rough) agreement of the values from the van Cittert–Zernike theorem (corresponding to a fully incoherent source) with our numerical values (for the partially coherent beam) is justified by the fact that *z* is large enough to lie in the *z*-range where the CL is linear [see discussion and Fig. 17 of Geloni *et al.* (2008 ▸)].

**Figure 6:**
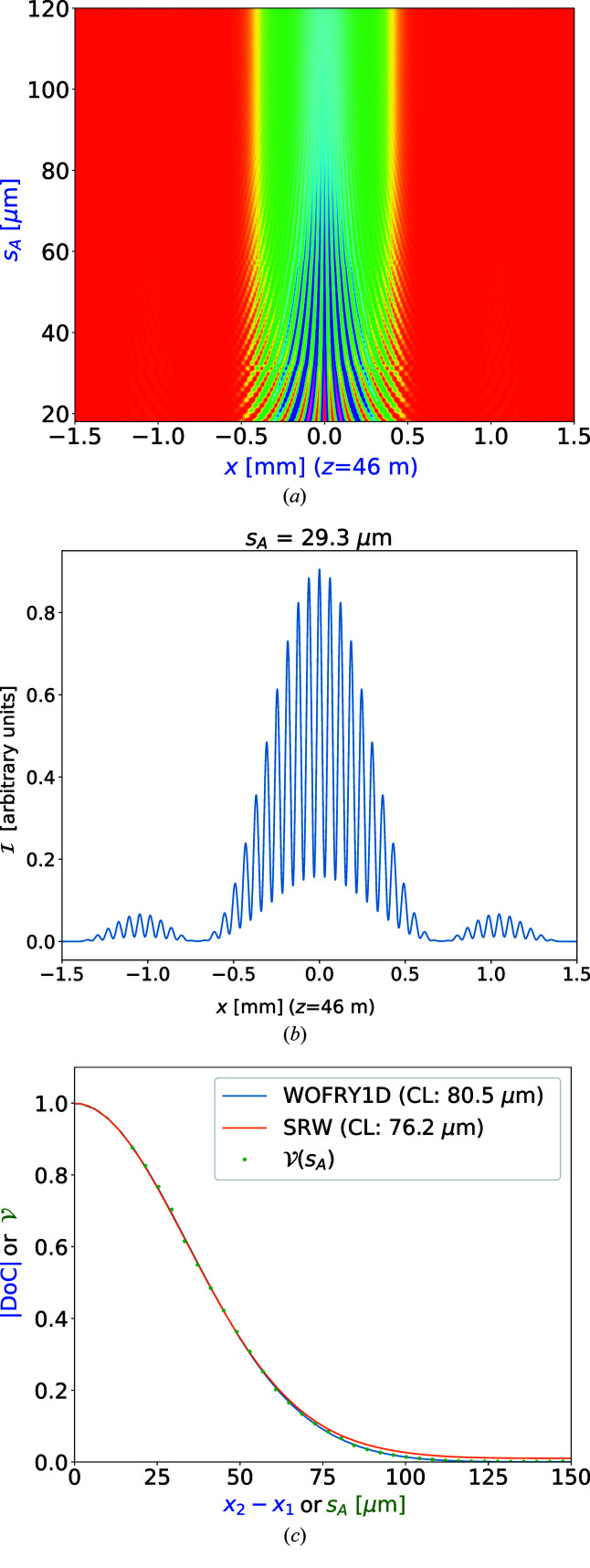
Simulation of beam propagation after a double-slit at 7 keV: (*a*) pattern intensity 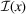 at *z* = 46 m versus the separation *s*_*A*_ between the slits. (*b*) Intensity profile 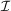 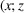 = 46 m) for slit separation *s* = 29.3 µm. (*c*) Modulus of the degree of coherence calculated from the profiles of Fig. 5 ▸ (top row), compared with the value obtained from the visibility extracted from (*a*).

It is worth mentioning that the modulus of the DoC (and therefore the CL) is obtained experimentally by measuring the interference of the two beams originated by a double-slit (Thompson & Wolf, 1957 ▸). Examples of this type of experiment with synchrotron radiation are presented elsewhere (Chang *et al.*, 2000 ▸; Paterson *et al.*, 2001 ▸; Leitenberger *et al.*, 2003 ▸; Tran *et al.*, 2005 ▸). We performed simulations with *WOFRY1D* placing two slits of 2.5 µm with horizontal separation *s*_*A*_ in the plane at *z* = 36 m, and propagating the resulting two beams to *z* = 46 m. The results are shown in Fig. 6 ▸(*a*), where it can be appreciated that the visibility of the fringes decreases when increasing *s*_*A*_. For a given *s*_*A*_, the intensity profile [*e.g.* Fig. 6 ▸(*b*)] is used to compute the visibility 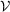 = 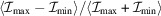 which is equal to the modulus of the DoC. We then obtained the visibility 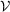 versus the slit separation *s*_*A*_ that gives (as expected) the same values as the modulus of DoC versus *x*_2_ − *x*_1_ in Fig. 5 ▸ [see Fig. 6 ▸(*c*)].

**Figure 7:**
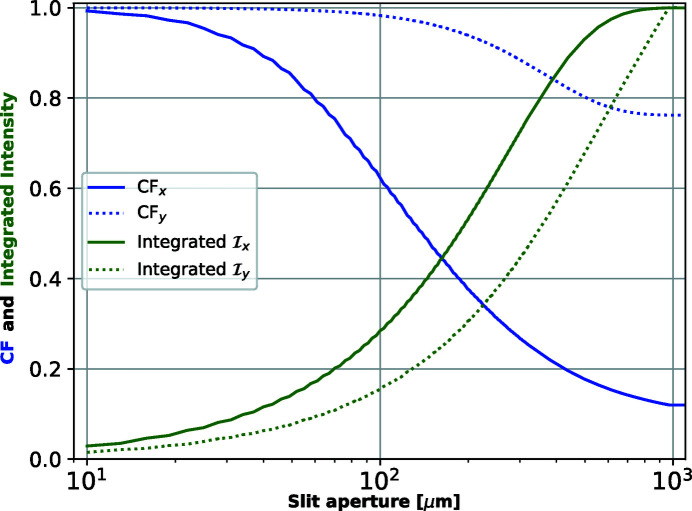
Coherent fraction (blue) and normalized integrated intensity (green) versus aperture for the horizontal (solid) and vertical (dotted) directions calculated by *WOFRY1D* at 7 keV.

The principal role of the slit is to control the beam coherent fraction. Closing the slit will increase the CF, with an obvious decrease in integrated intensity. The slit aperture necessary to obtain a ‘good’ coherence is somehow related to the CL, but quantitative values are better calculated using the CF versus the slit aperture. Within the CMD theory, this requires calculating coherent modes after the slit, which can be easily done with *WOFRY1D* (see Fig. 7 ▸). From the CF versus slit aperture plot, one can pick the aperture values to match the desired CF (we selected 90% or 70% values of CF to select slit apertures in Table 1 ▸), and at the same time estimate the losses in flux.

### 5.2. Image at sample position

**Figure 8:**
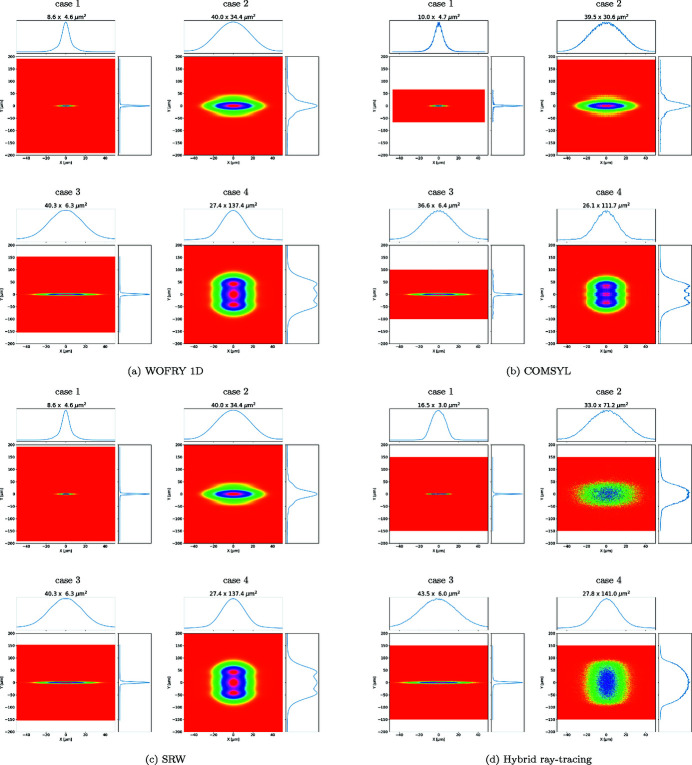
Calculations of the intensity distribution at the sample plane for the cases listed in Table 1 ▸.

The intensity distribution at the sample plane is displayed in Figs. 8 ▸(*a*)–8(*d*), for results using the *WOFRY1D*, *COMSYL*, *SRW-ME* and *HYBRID* codes. Beam dimensions are obtained by calculating the FWHM from the intensity distribution in one direction, resulting from integration along the other direction. They are displayed in the plots and summarized in Table 2 ▸. The results for case 1 show a horizontal profile mostly triangular with shoulders that evidence small diffraction fringes. The fringes are more resolved in the vertical direction. Case 2 presents in the horizontal a soft Gaussian-like profile, but in the vertical important symmetric shoulders are visible. Case 3 shows a smooth Gaussian profile in the horizontal and a small shoulder with fringes in the vertical. Case 4 shows a conventional smooth profile in the horizontal but an original three-lobe plateau in the vertical. This variety of profile distribution demonstrates how relevant the diffraction effects are, which modulate the beam shape in a non-trivial way.

The *WOFRY1D* results are shown in Fig. 8 ▸(*a*). The 1D intensity profile for each direction is obtained from the summation of several modes. High modes have very low eigenvalues. It is sufficient to consider only ten modes for accounting for more than 99% of the spectral density. The 2D intensity distribution shown in Fig. 8 ▸(*a*) is obtained by combining the calculated horizontal and vertical 1D profiles via the outer product. We can observe in the intensity distributions the same structures due to the diffraction effects as those observed with the other calculation methods. The beam profiles calculated, obtained with *COMSYL* and propagated with *WOFRY*, are shown in Fig. 8 ▸(*b*). *SRW-ME* results are given in Fig. 8 ▸(*c*). The good convergence of the values displayed is guaranteed by a convergence analysis described in Section S5. Hybrid ray-tracing for the four cases defined in Table 1 ▸ are shown in Fig. 8 ▸(*d*). The obtained intensity distributions are less structured than those calculated with the other wave-optics methods (*e.g.* the three-lobe plateau in case 4v is not reproduced). However, the FWHM values agree with full wave-optics calculations for most cases [except for two particular cases: 1h (*HYBRID* 17.3 µm, *WOFRY1D* 8.5 µm) and 2v (*HYBRID* 74.0 µm, *WOFRY1D* 32.4 µm)]. They will be discussed in the next section.

The agreement between the results of *WOFRY1D* in Fig. 8 ▸(*a*) and *SRW-ME* [Fig. 8 ▸(*c*)] is striking. All intensity distributions reproduce the same features, and the differences in FWHM values are less than 12%, a value that is compatible with the errors of the simulations. This result validates the 1D CMD method proposed here, whose requirements in computer power are extremely low (it runs very fast on an average laptop).

The numeric value for sizes calculated with the different methods (Table 2 ▸) depends not only on the code itself but also on the particular specific parameters in each method (number of pixels for sampling wavefronts, propagation parameters, *etc*.). To estimate the calculation error in the final size numbers, we vary randomly these specific parameters over a reasonable range (*e.g.* 10%). The dispersion (standard deviation) of the sizes obtained is a good estimator of the error in this parameter. This exercise would take considerable computational effort using 2D methods, but it can be easily done with *WOFRY1D*. We run 200 cases with 10% random variation in the number of pixels and zoom factor for drift spaces. The obtained sizes (horizontal × vertical) are 8.49 ± 0.60 µm × 4.97 ± 0.37 µm (case 1), 39.94 ± 2.98 µm × 32.77 ± 2.69 µm (case 2), 36.39 ± 2.89 µm × 6.12 ± 0.51 µm (case 3), and 24.18 ± 1.80 µm × 133.44 ± 10.06 µm (case 4). We confirmed that the values given in Fig. 8 ▸(*a*) are within these error intervals.

The calculated beam sizes should be completed with flux. At 7 keV, the undulator in the selected configuration emits a flux of 1.5 × 10^15^ photons s^−1^ (0.1% bandwidth)^−1^. Each of the three elements studied (slit, lens-1 and lens-2) absorbs part of the flux. The estimation of the absorption by the slit can be done using simple geometrical arguments, and the absorption by the lenses depends on the average Be thickness presented to the beam. The linear attenuation coefficient of Be at 7 keV is μ = 3 cm^−1^, giving 1.45% attenuation for a 50 µm-thick layer (like the lens thickness used in the simulations^7^). From the simulated data we extracted the absorption for the different absorbing elements (slit, lens-1 and lens-2) (see Table 3 ▸). We note a high absorption in lens-2 in cases 1 and 3; this is because lens-2 is over-illuminated, therefore the 1 mm physical aperture absorbs the beam considerably.

### 5.3. Computer resources

*COMSYL* requires high-performance computing to perform full CMD of the undulator beam, by solving the Friedholm problem and obtaining the full 2D eigenfunctions (coherent modes) and eigenvalues. The simulation of the source with *COMSYL* used to calculate Fig. 8 ▸(*b*) took 55 min using 28 × 3.30 GHz CPUs of 251.82 GB RAM, for getting 174 modes of 1691 × 563 pixels. The modes calculated by *COMSYL* were propagated with *WOFRY* in the *OASYS* environment (Rebuffi & Sanchez del Rio, 2017 ▸). The propagation used the 2D zoom propagator (see Section S3) and the optical elements described in Section 3.

The good convergence of the *SRW-ME* results in Fig. 8 ▸(*c*) is guaranteed by a convergence analysis described in Section S5 of the supporting information. It was used to determine the minimum number of electrons that produce accurate results. The *SRW-ME* simulations for the cases analyzed converge with only a few thousand electrons in a node with 28 CPUs totalizing 256 GB. This is because the beam after the slit has a relatively high CF.

Concerning running times, the simulations with the new *WOFRY1D* code run in a few seconds on a laptop. *HYBRID* also requires light computer resources and also runs on a laptop. The simulation of the full CMD with *COMSYL* required about 1 h with 28 cores. The source is then reused for propagating the different configurations. *SRW-ME* required a full source simulation for each configuration that also runs in about 1 h with 5000 electrons in a similar cluster.

### 5.4. Further simulations

The simulations presented, motivated by the EBSL1 project, use a simplified optical layout. They are used to validate the *WOFRY1D* tool before proceeding with further analyses.

A systematic and exhaustive study has been carried out using *WOFRY1D* for the new EBSL1 beamline also including other optical elements not considered here and multiple transfocator configurations. It is important to match correctly the two transfocators to guarantee that the focal point is located at the sample plane. Heat load deformation must be controlled at the white-beam mirrors and also at the monochromator. For that, the deformations calculated by finite-element methods are used in *WOFRY1D* for assessing the optical impact, as we did previously (Sanchez del Rio *et al.*, 2020 ▸). It is imperative to study the effect of mirror slope errors and surface errors at the lenses [as described by Celestre *et al.* (2020 ▸)]. These results will be described elsewhere.

## 6. Summary and conclusions

We presented in Section 2 the theory of partially coherence optics applied to undulator radiation and its implementation in two different solutions: (i) Monte Carlo multi-electron simulations as implemented in *SRW-ME* and (ii) coherent mode decomposition as implemented in *COMSYL* and in the new package *WOFRY1D*. The key point of *WOFRY1D* is that it needs very scarce computer resources. The factorization of the CSD in two directions (horizontal and vertical) is possible in many cases of major interest when the undulator is tuned close to odd-harmonic resonances, and when the horizontal and vertical emittances of the storage ring are not the same (*i.e.* non-round beams, as for the low-emittance storage ring EBS-ESRF). Section 3 summarizes the propagation of wavefronts along empty drift spaces and thin objects, which include the elements used in our simulations: slits and X-ray lenses.

We studied a particular case of focusing a partially coherent beam produced by an undulator in EBS-ESRF by a system of two transfocators (implemented as single parabolic lenses). This case is of interest to the new EBSL1 beamline being constructed at the ESRF. The combined effect of beam diffraction at the slit and global focusing by the two lenses produces images with a variety of intensity profiles (see Fig. 8 ▸). We included *HYBRID* ray-tracing results as this method can be used in the first simulation phase and produces approximated values of beam sizes and flux.

We have verified that simulations with the new *WOFRY1D* code are consistent with the other three simulation codes typically used to simulate synchrotron beamlines. A remarkable agreement is found between *WOFRY1D* and *SRW-ME* for the functions that describe partial coherence (CDS, DoC and CL) (see Figs. 4 ▸ and 5 ▸). We further used *WOFRY1D* to discuss the coherent fraction versus slit aperture (Fig. 7 ▸) and to verify that the modulus of the DoC is consistent with the results of a (simulated) experiment of two-beam interference (Fig. 6 ▸).

Partial coherence calculations using 2D wavefronts are expensive from the computation point of view, either because many thousands of wavefronts are propagated (like in *SRW-ME*) or because of the need to diagonalize an extremely large 4D cross-spectral function (like in *COMSYL*). The newly developed coherent mode decomposition uses 1D wavefronts and is very rapid and light. It can run interactively on a laptop. This opens new paths for intensive simulations of experiments using partially coherent beams, in particular for imaging applications or beamline optimization, where thousands of runs are necessary. It can also serve as a simulation engine for systems exploiting machine learning or for digital twins of beamline instruments.

The software tools developed here are available in the *WOFRY* add-on of the *OASYS* suite (Rebuffi & Sanchez del Rio, 2017 ▸). The *OASYS* workspaces and scripts for the simulations performed in this work are also available from https://github.com/oasys-esrf-kit/paper-multioptics-resources.

## 7. Related literature

The following references, not cited in the main body of the paper, have been cited in the supporting information: Chubar (2021 ▸); Chubar & Celestre (2019 ▸); Cowley & Moodie (1957 ▸); He *et al.* (2020 ▸); Pirro (2017 ▸); Schmidt (2010 ▸).

## Supplementary Material

Extension and detailed information of some sections. DOI: 10.1107/S1600577522008736/ay5600sup1.pdf

## Figures and Tables

**Table 1:** Configurations selected for 2D simulations The slit aperture (*a*_*x*_ and *a*_*y*_) is selected for obtaining CF_*x*_ = CF_*y*_ = 90% in cases 1 and 2, and CF_*x*_ = CF_*y*_ = 70% in cases 3 and 4.

Case h/v	*a*_*x*,*y*_ (µm)	*f*_1_ (m)	*f*_2_ (m)	*R*_1_ (µm)	*R*_2_ (µm)
1h	40.3	46.1	26.5	641.9	369.5
1v	227.0	15.0	22.2	209.4	309.6
2h	40.3	25.1	21.3	349.1	296.3
2v	227.0	42.2	55.6	588.6	775.3
3h	85.1	46.1	31.8	641.9	443.7
3v	506.7	85.2	27.8	1187.4	387.6
4h	85.1	25.1	20.7	349.1	288.7
4v	506.7	42.2	55.7	588.6	776.0

**Table 2:** Comparison of sizes (FWHM, in µm) calculated with different methods for the cases defined in Table 1 ▸ Numbers in brackets are the values for the fully coherent beam (single electron with *SRW*, first coherent mode with *COMSYL*/*WOFRY*, and zero emittance with *HYBRID*).

Case h/v	*WOFRY1D*	*COMSYL*	*SRW-ME*	*HYBRID*
1h	8.5 (8.1)	10.0 (9.9)	8.6 (7.5)	17.3 (17.5)
1v	4.8 (4.8)	4.7 (5.1)	4.6 (4.6)	3.3 (3.1)
2h	39.9 (38.2)	39.5 (39.5)	40.0 (36.1)	39.9 (37.5)
2v	32.4 (29.6)	30.6 (29.3)	34.4 (29.6)	74.0 (75.3)
3h	37.5 (29.0)	36.6 (28.1)	40.3 (28.4)	43.3 (33.0)
3v	6.1 (4.9)	6.4 (5.7)	6.3 (4.6)	6.5 (5.8)
4h	24.6 (19.1)	26.1 (18.6)	27.4 (18.0)	27.1 (18.8)
4v	133.7 (110.3)	111.7 (90.4)	137.4 (132.0)	150.2 (159.8)

**Table 3:** Comparison of beam intensity attenuation in percent by the slit, lens-1 and lens-2 for the partially coherent beam for the four cases studied The *WOFRY1D* data shown here come after combining the horizontal and vertical wavefronts using the outer product. Each profile corresponds to the intensity integrated along its perpendicular direction.

	Slit			Lens-1			Lens-2		
Case	*WOFRY1D*	*SRW-ME*	*HYBRID*	*WOFRY1D*	*SRW-ME*	*HYBRID*	*WOFRY1D*	*SRW-ME*	*HYBRID*
1	97.6	97.6	97.2	7.9	7.6	5.2	50.7	51.1	52.6
2	97.6	97.6	97.2	7.0	6.6	4.3	3.9	3.7	3.3
3	89.5	90.2	88.6	6.3	6.1	4.6	23.4	21.8	25.2
4	89.5	90.2	88.6	8.0	7.7	6.2	3.8	3.6	3.6

## Footnotes

1 At third-generation light sources, very restrictive pinholes and slits were used for spatial filtering with a dramatic loss of flux when improving and tuning the coherent fraction of the beam. Due to the increased coherent fraction at fourth-generation sources, much less restrictive filtering is necessary, with higher photon transmission as a consequence.

2 Geloni *et al.* (2008 ▸) provide a counter-example discussing the high-resolution monochromator of their reference 15.

3 During the review of this manuscript, the authors came across the work of Trebushinin *et al.* (2022 ▸), which also covers the theoretical background of undulator statistical properties.

4 Spectral density is sometimes loosely called intensity. The spectral density and the intensity functions are equivalent for the stationary case. The same holds for the CSD and the mutual optical intensity (MOI) (Mandel & Wolf, 1995 ▸).

5 It is sometimes called complex spatial (or spectral) degree of coherence, see §4.3.2 in Mandel & Wolf (1995 ▸).

6 Throughout this work we have used the following values of electron beam sizes at the centre of the straight section: σ_*x*_ = 29.7 µm,  = 4.37 µrad, σ_*y*_ = 5.29 µm,  = 1.89 µrad, corresponding to beam emittances: ε_*x*_ = 130 pm rad, ε_*y*_ = 10 pm rad, and beta functions β_*x*_ = 6.8 m, β_*y*_ = 2.8 m.

7 In the simulations, the horizontal and vertical focusing are separated in two lenses, with accumulated thickness 100 µm thus absorption 3%.

## References

[bb1] Agarwal, G. S. & Simon, R. (2000). *Opt. Lett.* **25**, 1379–1381. 10.1364/ol.25.001379 18066223

[bb2] Bazarov, I. V. (2012). *Phys. Rev. ST Accel. Beams*, **15**, 050703.

[bb3] Canestrari, N., Chubar, O. & Reininger, R. (2014). *J. Synchrotron Rad.* **21**, 1110–1121. 10.1107/S1600577514013058 25178000

[bb4] Celestre, R., Berujon, S., Roth, T., Sanchez del Rio, M. & Barrett, R. (2020). *J. Synchrotron Rad.* **27**, 305–318. 10.1107/S1600577519017235 PMC7842213 32153269

[bb5] Chang, C., Naulleau, P., Anderson, E. & Attwood, D. (2000). *Opt. Commun.* **182**, 25–34.

[bb690] Chubar, O. (2021). *SRW*, https://www.github.com/ochubar/SRW.

[bb6] Chubar, O., Berman, L., Chu, Y. S., Fluerasu, A., Hulbert, S., Idir, M., Kaznatcheev, K., Shapiro, D., Shen, Q. & Baltser, J. (2011). *Proc. SPIE*, **8141**, 814107.

[bb691] Chubar, O. & Celestre, R. (2019). *Opt. Express*, **27**, 28750–28759. 10.1364/OE.27.028750 31684620

[bb7] Chubar, O. & Elleaume, P. (1998). *Proceedings of the Sixth European Particle Accelerator Conference (EPAC98)*, 22–26 June 1998, Stockholm, Sweden, pp. 1177–1179. THP01G.

[bb8] Cloetens, P., Barrett, R., Baruchel, J., Guigay, J.-P. & Schlenker, M. (1996). *J. Phys. D Appl. Phys.* **29**, 133–146.

[bb692] Cowley, J. M. & Moodie, A. F. (1957). *Acta Cryst.* **10**, 609–619.

[bb9] Dejus, R. J. & Luccio, A. (1994). *Nucl. Instrum. Methods Phys. Res. A*, **347**, 61–66.

[bb10] Gasbarro, A. & Bazarov, I. (2014). *J. Synchrotron Rad.* **21**, 289–299. 10.1107/S1600577514000277 24562550

[bb11] Geloni, G., Saldin, E., Schneidmiller, E. & Yurkov, M. (2008). *Nucl. Instrum. Methods Phys. Res. A*, **588**, 463–493.

[bb12] Glass, M. (2017*a*). *COMSYL GitHub repository*, https://www.github.com/oasys-kit/comsyl.

[bb13] Glass, M. (2017*b*). *Statistical optics for synchrotron emission: numerical calculation of coherent modes.* Phd thesis, Université Grenoble Alpes, France (https://tel.archives-ouvertes.fr/tel-01664052).

[bb14] Glass, M. & Sanchez del Rio, M. (2017). *Europhys. Lett.* **119**, 34004.

[bb15] Goodman, J. W. (2017). *Introduction to Fourier Optics*, 4th ed. W. H. Freeman and Company.

[bb693] He, A., Chubar, O., Rakitin, M., Samoylova, L., Fortmann-Grote, C., Yakubov, S. & Buzmakov, A. (2020). *Proc. SPIE*, **11493**, 114930H.

[bb16] Hernandez, V., Roman, J. E. & Vidal, V. (2005). *ACM Trans. Math. Softw.* **31**, 351–362.

[bb17] Hirschmugl, C. J., Sagurton, M. & Williams, G. P. (1991). *Phys. Rev. A*, **44**, 1316–1320. 10.1103/physreva.44.1316 9906081

[bb18] Jackson, J. D. (1999). *Classical Electrodynamics.* New York: Wiley.

[bb19] Kelly, D. P. (2014). *J. Opt. Soc. Am. A*, **31**, 755. 10.1364/JOSAA.31.000755 24695137

[bb20] Kim, K.-J. (1986). *Proc. SPIE*, **0582**, 2–9.

[bb21] Laundy, D., Alcock, S. G., Alianelli, L., Sutter, J. P., Sawhney, K. J. S. & Chubar, O. (2014). *Proc. SPIE*, **9209**, 920903.

[bb22] Leitenberger, W., Wendrock, H., Bischoff, L., Panzner, T., Pietsch, U., Grenzer, J. & Pucher, A. (2003). *Physica B*, **336**, 63–67.

[bb23] Li, K. & Jacobsen, C. (2015). *J. Opt. Soc. Am. A*, **32**, 2074–2081. 10.1364/JOSAA.32.002074 26560922

[bb24] Li, K., Wojcik, M. & Jacobsen, C. (2017). *Opt. Express*, **25**, 1831–1846. 10.1364/OE.25.001831 29519036

[bb25] Li, R. & Chubar, O. (2022). *Opt. Express*, **30**, 5896–5915. 10.1364/OE.452247 35209542

[bb26] Lordano, S. (2022). Private communication.

[bb27] Mandel, L. & Wolf, E. (1995). *Optical Coherence and Quantum Optics.* Cambridge University Press.

[bb28] Munro, P. R. T. (2019). *J. Opt. Soc. Am. A*, **36**, 1197–1208. 10.1364/JOSAA.36.001197 31503958

[bb29] Nash, B., Goldring, N., Edelen, J., Webb, S. & Celestre, R. (2021). *Phys. Rev. Accel. Beams*, **24**, 010702.

[bb30] Paganin, D. M. (2006). *Coherent X-ray Optics.* Oxford University Press.

[bb31] Paganin, D. M. & Sanchez del Río, M. (2019). *Phys. Rev. A*, **100**, 043813.

[bb32] Paterson, D., Allman, B. E., McMahon, P. J., Lin, J., Moldovan, N., Nugent, K. A., McNulty, I., Chantler, C. T., Retsch, C. C., Irving, T. H. K. & Mancini, D. C. (2001). *Opt. Commun.* **195**, 79–84.

[bb694] Pirro, G. (2017). *Application of Scaled Wave Optics Propagator to Model Synchrotron Beamlines.* MSc thesis, Politecnico di Milano, Italy.

[bb33] Raimondi, L. & Spiga, D. (2015). *Astron. Astrophys.* **573**, A22.

[bb34] Rebuffi, L. & Sánchez del Río, M. (2016). *J. Synchrotron Rad.* **23**, 1357–1367. 10.1107/S1600577516013837 PMC5298219 27787241

[bb35] Rebuffi, L. & Sanchez del Rio, M. (2017). *Proc. SPIE*, **10388**, 103880S.

[bb36] Rebuffi, L. & Shi, X. (2020). *Proc. SPIE*, **11493**, 1149303.

[bb37] Rees, W. G. (1987). *Eur. J. Phys.* **8**, 44–48.

[bb38] Sanchez del Rio, M., Canestrari, N., Jiang, F. & Cerrina, F. (2011). *J. Synchrotron Rad.* **18**, 708–716. 10.1107/S0909049511026306 PMC3267628 21862849

[bb39] Sanchez del Rio, M., Celestre, R., Glass, M., Pirro, G., Herrera, J. R., Barrett, R., da Silva, J. C., Cloetens, P., Shi, X. & Rebuffi, L. (2019). *J. Synchrotron Rad.* **26**, 1887–1901. 10.1107/S160057751901213X 31721731

[bb40] Sanchez del Rio, M., Wojdyla, A., Goldberg, K. A., Cutler, G. D., Cocco, D. & Padmore, H. A. (2020). *J. Synchrotron Rad.* **27**, 1141–1152. 10.1107/S1600577520009522 32876588

[bb695] Schmidt, J. D. (2010). *Numerical Simulation of Optical Wave Propagation.* SPIE Press.

[bb41] Shi, X., Reininger, R., Harder, R. & Haeffner, D. (2017). *Proc. SPIE*, **10388**, 10388.

[bb42] Shi, X., Reininger, R., Sanchez del Rio, M. & Assoufid, L. (2014). *J. Synchrotron Rad.* **21**, 669–678. 10.1107/S160057751400650X PMC4861879 24971960

[bb43] Stern, A. & Javidi, B. (2004). *Opt. Eng.* **43**, 239–250.

[bb44] Tanaka, T. (2014). *Phys. Rev. ST Accel. Beams*, **17**, 060702.

[bb45] Tanaka, T. & Kitamura, H. (2001). *J. Synchrotron Rad.* **8**, 1221–1228. 10.1107/s090904950101425x 11679776

[bb46] Thery, S., Glass, M. & Sanchez del Rio, M. (2016). *PySRU GitHub repository*, https://www.github.com/oasys-kit/pySRU, https://github.com/oasys-kit/pySRU.

[bb47] Thompson, B. J. & Wolf, E. (1957). *J. Opt. Soc. Am.* **47**, 895–902.

[bb48] Tran, C. Q., Peele, A. G., Roberts, A., Nugent, K. A., Paterson, D. & McNulty, I. (2005). *Opt. Lett.* **30**, 204–206. 10.1364/ol.30.000204 15675714

[bb49] Trebushinin, A., Geloni, G., Rakshun, Y. & Serkez, S. (2022). *Optica*, **9**, 842–852.

[bb50] Vaughan, G. B. M., Wright, J. P., Bytchkov, A., Rossat, M., Gleyzolle, H., Snigireva, I. & Snigirev, A. (2011). *J. Synchrotron Rad.* **18**, 125–133. 10.1107/S0909049510044365 PMC3267637 21335897

[bb51] Walker, R. P. & Diviacco, B. (1992). *Rev. Sci. Instrum.* **63**, 392–395.

[bb52] Wiedemann, H. (2019). *Particle Accelerator Physics*, 4th ed. Springer International Publishing.

[bb53] Xu, H., Zhu, Z., Li, X., Liu, P., Dong, Y. & Zhou, L. (2022). *Opt. Express*, **30**, 7625–7635. 10.1364/OE.448337 35299520

[bb54] Zhang, W., Zhang, H. & Jin, G. (2020). *Opt. Express*, **28**, 39916–39932. 10.1364/OE.413636 33379530

