# Supplementary material for: A fast and lightweight tool for partially coherent beamline simulations in fourth-generation storage rings based on coherent mode decomposition
Source: J Synchrotron Radiat. 2022 Oct 18;29(Pt 6):1354–67. doi: 10.1107/S1600577522008736 (PMC9641568; doi:10.1107/S1600577522008736)
Supplement: Supplementary file 1 [file s-29-01354-sup1.pdf]

JOURNAL OF SYNCHROTRON RADIATION

Volume 29 (2022)

Supporting information for article:

A fast and lightweight tool for partially coherent beamline simulations in fourth-generation storage rings based on coherent mode decomposition

Manuel Sanchez del Rio, Rafael Celestre, Juan Reyes-Herrera, Philipp Brumund and Marco Cammarata

S1. Wavefront propagation in free-space

Under the scalar theory, a generic wavefront obeying the wave-equation and completely described at a position z, that is, $E_\omega(\mathbf{r})$ known for all the $xy-$plane will propagate (evolve) between two parallel planes separated by a distance $L = z' - z$ as:

$$E_\omega(\mathbf{r}') = \frac{k}{2\pi i} \int\limits_{\Sigma} E_\omega(\mathbf{r}) \frac{\exp\left(ik|\mathbf{r}' - \mathbf{r}|\right)}{|\mathbf{r}' - \mathbf{r}|} \cos\theta \; \mathrm{d}\mathbf{s}. \tag{1}$$

Equation (1) is the first Rayleigh-Sommerfeld diffraction equation (Huygens-Fresnel principle) and is valid for the case where $|\mathbf{r}' - \mathbf{r}| \gg \lambda$, with $\lambda = 2\pi/k$. We define a normal vector parallel to the optical axis ($z-$axis) $\vec{\ell}$ so that θ is the angle between $-\vec{\ell}$ and the vector $\mathbf{r}' - \mathbf{r}$; Σ is the $xy-$plane in z where the integration takes place with $\mathrm{d}\mathbf{s} = \mathrm{d}x\mathrm{d}y$. Further simplification to equation (1) can be done using the paraxial approximation. In this case, it is assumed that $\cos\theta \approx 1$; and that the term $|\mathbf{r}' - \mathbf{r}| = \sqrt{(x' - x)^2 + (y' - y)^2 + L^2}$ can be expanded in a Taylor series with $L^2 \gg (x' - x)^2$ and $L^2 \gg (y' - y)^2$. Retaining the quadratic term in the exponential function, but dropping it for the denominator:

$$\begin{aligned} E_\omega(\mathbf{r}') =& \frac{k \exp\left(ikL\right)}{2\pi i L} \cdot \\ & \cdot \int\limits_{\Sigma} E_\omega(\mathbf{r}) \exp\left\{ \frac{ik}{2L} \left[(x' - x)^2 + (y' - y)^2\right] \right\} \mathrm{d}\mathbf{s}. \end{aligned} \tag{2}$$

This approximation is known as the Fresnel diffraction integral.

S2. The SRW propagators

The SRW propagators are grouped into three main methods for 2D wavefront propagation (Chubar, 2021). The first main method lies under the "standard Fresnel propagator", which can be implemented as (a) direct numerical calculation of the convolution integral in equation (2) by means of nested Riemann summations; or (b) through the application of the convolution theorem:

$$\begin{aligned} E_\omega(\mathbf{r}') &= \frac{k \exp(ikL)}{2\pi i L} E_\omega(\mathbf{r}) * \exp\left\{\frac{ik}{2L}\left[(x')^2 + (y')^2\right]\right\} \\ &= \exp(ikL)\mathcal{F}^{-1}\big\{\mathcal{F}\{E_\omega(\mathbf{r})\}\mathcal{F}\{h(\mathbf{r}')\}\big]\big\} \\ &= \exp(ikL)\mathcal{F}^{-1}\big\{\mathcal{F}\{E_\omega(\mathbf{r})\}\exp\big[-i\pi\lambda L\big(f_x^2 + f_y^2\big)\big]\big\}, \end{aligned} \tag{3}$$

where $\mathcal{F}\{\bullet\}$ is the two-dimensional Fourier transform (FT) and $\mathcal{F}^{-1}\{\bullet\}$ denotes inverse FT. This second approach is efficiently implemented in SRW using only two fast Fourier transforms (FFTs) because the kernel $h(\mathbf{r}')$ has an analytical Fourier transform. Downsides to the FFT-based implementation include the heavy sampling needed to avoid aliasing and also necessary in order to resolve small features in the propagated wavefront $E_\omega(\mathbf{r}')$, since the application of equation (3) limits the range and sampling in the output plane to those of the input plane $E_\omega(\mathbf{r})$ (Kelly, 2014). The interest in having a direct- and a reciprocal-space implementation of equation (2) is summarised in Fig. 1 from (Li & Jacobsen, 2015).

A second family of propagators is obtained by the analytical treatment of the quadratic radiation phase term in equation (2), which allows for considerable economy of memory and CPU resources as compared to the standard Fresnel free-space propagator (Chubar & Celestre, 2019). Without losing generality, we assume that the electric field $E_\omega(\mathbf{r})$ has a quadratic phase term defined by the wavefront curvature radii (R_x, R_y) centred at (x_0, y_0):

$$E_\omega(\mathbf{r}) = F_\omega(\mathbf{r}) \exp\left\{\frac{ik}{2}\left[\frac{(x - x_0)^2}{R_x} + \frac{(y - y_0)^2}{R_y}\right]\right\}. \tag{4}$$

Plugging equation (4) into equation (2) and collecting terms:

$$
\begin{aligned}
&E_\omega(\mathbf{r}') = \\
&\frac{k\exp{(ikL)}}{2\pi iL}\exp\left\{\frac{ik}{2}\left[\frac{(x'-x_0)^2}{R_x+L}+\frac{(y'-y_0)^2}{R_y+L}\right]\right\}\cdot \\
&\cdot\int\limits_{\Sigma} F_\omega(\mathbf{r})\exp\left\{\frac{ik}{2L}\left[\frac{R_x+L}{R_x}\left(\frac{R_x x'+Lx_0}{R_x+L}-x\right)^2+\right.\right. \\
&\qquad\qquad\left.\left.+\frac{R_y+L}{R_y}\left(\frac{R_y y'+Ly_0}{R_y+L}-y\right)^2\right]\right\}\,\mathrm{d}\mathbf{s}.
\end{aligned}
\tag{5}
$$

Much like equation (2), equation (5) is a convolution type integral that not only can be computed using the convolution theorem, but also has an analytical Fourier transform of its kernel. We draw attention to the fact that the convolution is done regarding scaled coordinates:

$$
\hat{\mathbf{r}} = (\hat{x},\hat{y}) = \left(\frac{R_x x'+Lx_0}{R_x+L},\frac{R_y y'+Ly_0}{R_y+L}\right), \tag{6}
$$

Equation 5 can, then, be calculated as:

$$
\begin{aligned}
&E_\omega(\mathbf{r}') = \exp{(ikL)}\cdot \\
&\cdot\exp\left\{\frac{ik}{2}\left[\frac{(x'-x_0)^2}{R_x+L}+\frac{(y'-y_0)^2}{R_y+L}\right]\right\}\sqrt{\frac{R_xR_y}{(R_x+L)(R_y+L)}}\cdot \\
&\cdot\mathcal{F}^{-1}\left\{\mathcal{F}\{F_\omega(\mathbf{r})\}\exp\left[-i\pi\lambda L\left(\frac{R_x}{R_x+L}f_x^2+\frac{R_y}{R_y+L}f_y^2\right)\right]\right\},
\end{aligned}
\tag{7}
$$

which is of particular interest because the application of the convolution theorem implemented with FFTs yields a natural rescaling of the ranges of the output plane [see Fig. 1 in (Chubar & Celestre, 2019)]. Padding with zeros and resampling the input field in order to obtain reasonable results in the output plane are less often necessary than when dealing with the formulation in equation (3). This propagator, by far the most versatile in SRW, is presented to the user as two separate methods that differ on the estimation of the wavefront curvature R_x and R_y and on the processing near the beam waist ($R_x \approx -L$ and $R_y \approx -L$).

Two less general propagators form the third family of methods proposed by SRW. The first one is based on the Fraunhofer approximation of equation (2) and is used

for wavefront propagation over a very large distance (far field):

$$
\begin{aligned}
E_\omega(\mathbf{r}') =& \frac{k \exp(ikL)}{2\pi i L} \exp\left[i \frac{k}{2L}(x'^2 + y'^2)\right] \cdot \\
& \cdot \int_\Sigma E_\omega(\mathbf{r}) \exp\left[- i \frac{2\pi}{\lambda L}(x'x + y'y)\right] \mathrm{d}\mathbf{s}.
\end{aligned}
\tag{8}
$$

The integral in equation (8) is a Fourier transform of the field $E_\omega(\mathbf{r})$ with spatial frequencies given by $f_x = x'/\lambda L$ and $f_y = y'/\lambda L$. Its implementation in SRW is done using a single FFT. A second propagator based on a single FFT is implemented to cover the case of a focusing wavefront being propagated over some distance to the beam waist. A converging wavefront written as $E_\omega(\mathbf{r}) = F_\omega(\mathbf{r}) \cdot \exp[-i\frac{k}{2q}(x^2 + y^2)]$ plugged into equation (2) yields:

$$
\begin{aligned}
& E_\omega(\mathbf{r}') = \\
& \frac{k \exp(ikL)}{2\pi i L} \exp\left[i \frac{k}{2L}(x'^2 + y'^2)\right] \cdot \\
& \quad \cdot \int_\Sigma F_\omega(\mathbf{r}) \exp\left[- i \frac{2\pi}{\lambda L}(x'x + y'y) + \right. \\
& \qquad\qquad \left. + i \frac{k}{2L}(x^2 + y^2) - i \frac{k}{2q}(x^2 + y^2)\right] \mathrm{d}\mathbf{s},
\end{aligned}
\tag{9}
$$

where q is the distance from the input plane to the beam waist. When the wavefront is propagated to the the image plane, that is, $L = q$, the integral in equation (9) assumes the formalism of a Fourier transform with $f_x = x'/\lambda q$ and $f_y = y'/\lambda q$.

S3. The WOFRY propagators

The WOFRY propagators can be used to propagate any arbitrary wavefront generated within this software and in particular, the 1D and 2D coherent modes described in the main paper. Like SRW, WOFRY offers the standard Fresnel propagator using 2 FFTs [equation (3)] and the Fraunhofer approximation calculated with one FFT [equation (8)]. Both propagators are available in 1D and 2D implementations. To overcome the issues with the output plane range when applying the convolution theorem for the Fresnel propagator, WOFRY offers an implementation based on works by Schmidt (2010) and Pirro (2017) that permits scale of the output plane range while retaining the possibility of calculation by means of the convolution theorem. Let m_x and m_y be magnification factors for the output plane range and:

$$\begin{aligned}(x'-x)^2 &= m_x\left(\frac{x'}{m_x}-x\right)^2+\left(\frac{m_x-1}{m_x}\right)x'^2+(1-m_x)x^2\\ (y'-y)^2 &= m_y\left(\frac{y'}{m_y}-y\right)^2+\left(\frac{m_y-1}{m_y}\right)y'^2+(1-m_y)y^2,\end{aligned} \tag{10}$$

that we use in equation (2):

$$\begin{aligned}&E_\omega(\mathbf{r}') =\\ &\frac{k\exp(ikL)}{2\pi iL}\exp\left\{i\frac{k}{2L}\left[\left(\frac{m_x-1}{m_x}\right)x'^2+\left(\frac{m_y-1}{m_y}\right)y'^2\right]\right\}\\ &\int_\Sigma F_\omega(\mathbf{r})\exp\left\{-i\frac{k}{2L}\left[m_x\left(\frac{x'}{m_x}-x\right)^2+m_y\left(\frac{y'}{m_y}-y\right)^2\right]\right\}\,\mathrm{d}\mathbf{s}\end{aligned} \tag{11}$$

with:

$$E_\omega(\mathbf{r}) = F_\omega(\mathbf{r})\exp\left\{i\frac{k}{2L}\left[(1-m_x)x^2+(1-m_y)y^2\right]\right\}. \tag{12}$$

Equation (11) is a convolution between $F_\omega(\mathbf{r})$ and a kernel with reduced scaled $\hat{\mathbf{r}} = (\hat{x},\hat{y}) = \left(x'/m_x, y'/m_y\right)$. This kernel has an analytical Fourier transform and the

application of the convolution theorem with two FFTs is possible:

$$
\begin{aligned}
&E_\omega(\mathbf{r'}) = \\
&\frac{\exp{(ikL)}}{\sqrt{m_x m_y}} \exp\left\{i\frac{k}{2L}\left[\left(\frac{m_x-1}{m_x}\right)x'^2 + \left(\frac{m_y-1}{m_y}\right)y'^2\right]\right\}\cdot \\
&\qquad \cdot \mathcal{F}^{-1}\left\{\mathcal{F}\{F_\omega(\mathbf{r})\}\exp\left[-i\pi\lambda L\left(\frac{f_x^2}{m_x}+\frac{f_y^2}{m_y}\right)\right]\right\}.
\end{aligned} \tag{13}
$$

Note that when $m_x = 1$ and $m_y = 1$ we recover equation (2) and equation (3). A 1D version of this "zoom" propagator is also available in WOFRY.

For the cases where accuracy should be privileged over execution time, a 1D paraxial version of the Rayleigh-Sommerfeld integral where $\cos(\theta) = 1$ is also implemented in WOFRY. Similarly to the direct numerical calculation of the Fresnel diffraction integral, the 1D version of equation (1) is implemented as a Riemann summation (Sanchez del Rio *et al.*, 2020).

S4. Transmission Elements

Consider an arbitrary-shaped scattering volume. Suppose that such scatterer is completely confined within a region $z_0 \leq z \leq z_1$ and outside that there is vacuum. Let this sample be illuminated by a plane-wave moving along the positive direction of the optical axis ($z-$axis). In the absence of the scatterer, the gradient between the $z = z_0$ and $z = z_1$ planes is very well defined and parallel to the optical axis. It follows [§2.2 in (Paganin, 2006)] that if the scatterer is sufficiently weak as to minimally disturb the path that the wave-field would have taken in its absence, the transmission of a wave-field through this sample is given by:

$$E_\omega(x, y, z_1) \approx \exp\left\{ -\frac{ik}{2} \int\limits_{z=z_0}^{z=z_1} \left[1 - n_\omega^2(x, y, z)\right] \,\mathrm{d}z \right\} E_\omega(x, y, z_0). \tag{14}$$

Equation (14) shows that the effect of a weak scatterer can be accounted for by a multiplicative complex transmission element represented by the complex exponential. In the X-ray regime the index of refraction is typically very close to unity and often expressed as $n_\omega = 1 - \delta_\omega + i \cdot \beta_\omega$, which allows for the approximation $1 - n_\omega(x, y, z)^2 \approx 2\left[\delta_\omega(x, y, z) - i \cdot \beta_\omega(x, y, z)\right]$ that can be substituted in equation (14). The $z-$dependence of δ_ω and β_ω is abandoned in the projection approximation, hence the complex transmission element in equation (14) can be reduced to:

$$\begin{aligned} \mathrm{T}(x, y, z) &= \exp\left\{ -ik \int\limits_{z=z_0}^{z=z_1} \left[\delta_\omega(x, y) + i \cdot \beta_\omega(x, y)\right] \,\mathrm{d}z \right\} \\ &= \exp\left\{ -ik\left[\delta_\omega(x, y) + i \cdot \beta_\omega(x, y)\right] \Delta_z(x, y) \right\}. \end{aligned} \tag{15}$$

Δ_z is the projected thickness along the $z-$axis and it depends on the transverse coordinates (x, y), which can be dropped out for a more concise representation.

For the cases where the projection approximation may not be adequate to correctly represent the scattering volume in question, multi-slicing techniques[1] can be used for

[1] This technique was first described in the context of the scattering of electrons by atoms and crystals (Cowley & Moodie, 1957).

describing the wave-field propagation inside an arbitrary-shaped scattering volume [see discussion in §2.7 in (Paganin, 2006), (Li *et al.*, 2017) and (Munro, 2019)]. Consider again an arbitrary-shaped scattering volume. If its presence considerably disturbs the path that the wave-field would have taken in its absence. It is possible to section the sample into a number N of parallel slabs until the projection approximation is valid between two adjacent slices. The projected thickness Δ_z to be used in equation (15) is the one in between slices, which are $\Delta_S = (z_1 - z_0)/N$ apart from each other. Each slice represented as a thin element in projection approximation is separated by vacuum. The propagation of a wave-field through this sample is done step-wise, where each step is composed of multiplication by a complex transmission element in projection approximation and a free-space propagation over a distance Δ_S. The output field from this operation is again multiplied by a complex transmission element in projection approximation followed by a free-space propagation from the plane ψ_j to the ψ_{j+1}. This operation is done recursively N times until the wave-field emerges from the sample.

S5. Some considerations on partially-coherent calculations using SRW's macro-electrons & simulation convergence

The convergence of the SRW-ME method is based on the *finesse* with which the distribution $f(\mathcal{S})$ is sampled (Eq. 4 in the main paper). While an exquisitely large number of *me's* will lead to a more accurate simulation, the resulting calculation would be very long and impossible to be performed on personal computers within a reasonable time even if performed in parallel. The number of *me's* depends on the overall beamline degree of coherence at the observation plane, which is impacted by the source coherent fraction and beamline overall transmission (e.g. slits, creation of secondary sources or any other spatial filtering scheme). Special attention to the number of macro-electrons should be given if the simulation accounts for vibrations in the beamline elements or broad-band radiation (e.g. pink beam or radiation filtered by multi-layer monochromators).

To illustrate the effect of the number of macro-electrons on the beam profile we choose the studied cases 1 and 3 from the section 5 in the main paper - due to their CF, cases 2 and 4 are expected to have the same convergence as 1 and 3, respectively. Both systems 1 & 3 (and 2 & 4) have the same X-ray source and are illuminated up until the slits (36 m downstream U18) by the same beam, differing mainly by the coherent fraction selected for the rest of the beamline with case 1 having a higher CF than case 3 - refer to Table 1 for the complete simulation parameters. The results for a selected number of *me's* are shown in Figs. S1 and S2. The 1 *me* simulation represents the filament-beam source, where the electron beam emittance is negligible and the fully spatial coherence is assumed - this is often called a "single electron simulation". On the other extreme, an exaggerated value of 100k $me's$ is chosen as a way of guaranteeing convergence by brutal force. Two criteria are used to evaluate the convergence of the simulations: the beam shape and peak intensity stabilisation. The profile cuts in

Fig. S1a-b and Fig. S2a-b show that the profile shape starts to converge to that of a 100k $me's$ after ∼500 macroelectrons for case 1 and ∼1k macroelectrons for case 3. Beyond that, it is necessary to resort to the relative error standard deviation and the peak intensity stabilisation. Fig. S1c-d and Fig. S2c-d show that for both cases, the convergence happens between 2k and 5k $me's$. Further increase in the number of macro electrons does not translate into improvements in the simulations (see simulations for 10k $me's$ onward), but increase greatly the cost of the calculation as shown in Fig. S3. For the work presented here a good compromise between accuracy and efficiency of the calculations is reached at 5k $me's$. Other factors contributing to the total elapsed simulation time and overall parallel performance of SRW-ME method are presented in §3.3 from (He *et al.*, 2020), but these do not impact the SRW-ME convergence.

It is important to note that this large scan procedure is merely illustrative. Usually, an experienced optical designer starts with a good guess of the necessary number of $me's$ based on the characteristics of the source (degree of coherence) and optical system (transmission and expected degree of coherence at the observation plane). This choice usually includes considerations of time and resource consumption. If there are signs that the choice may be too low, further attempts with higher $me's$ should be done. If the simulation looks fine from the first guess, reducing the number of $me's$ is also interesting as very often it is necessary to repeat the simulations (eg. testing different configurations, different energies, tolerancing or even different observation planes). At the time of writing, the authors are unaware of any widespread metric within the SRW's community capable of giving the exact number of *me's* necessary for the convergence of the SRW-ME method other than the $me's$ scan. We welcome the discussion on SRW-ME convergence and we encourage the reader to reach out if they employ any interesting and reproducible convergence metric that is less time (and resource) consuming.

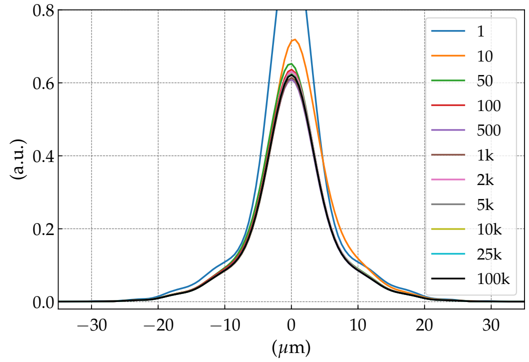

(a) horizontal cut at $y = 0$

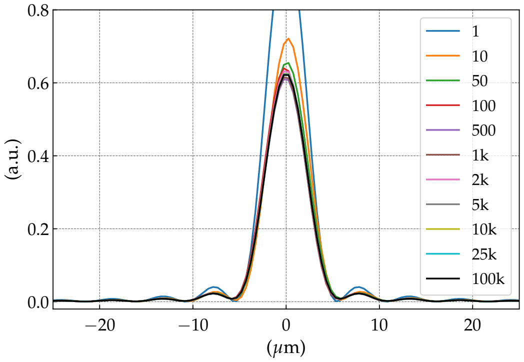

(b) vertical cut at $x = 0$

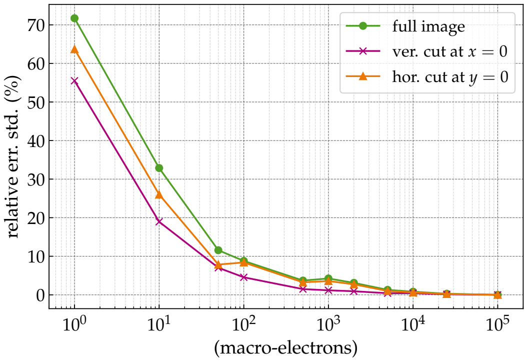

(c) relative error std. deviation

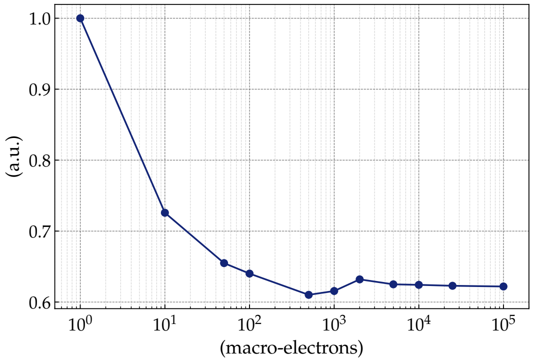

(d) peak intensity

Fig. S1. Partially-coherent simulations convergence study: case 1. (a) horizontal and (b) vertical intensity cuts at E=7 keV for $me's$ ranging from 1 to 100k. (c) errors relative to the $me's$ =100k plots and (d) peak intensity.

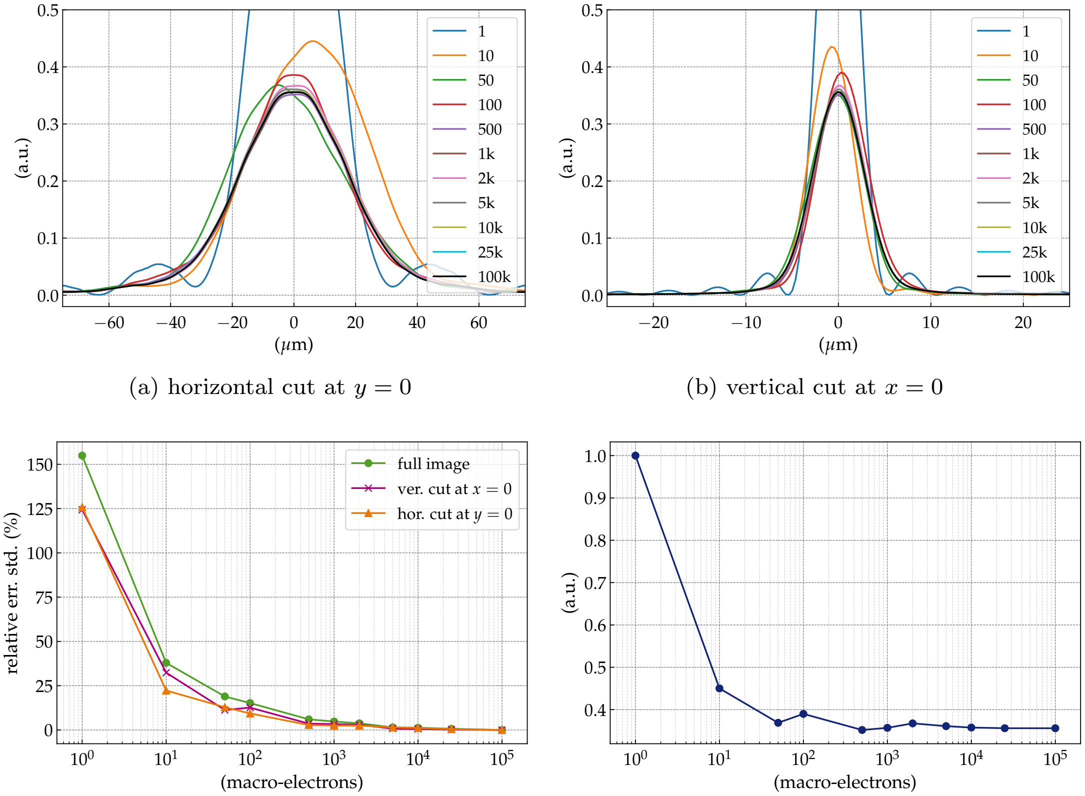

(a) horizontal cut at $y = 0$

(b) vertical cut at $x = 0$

(c) relative error std. deviation

(d) peak intensity

Fig. S2. Partially-coherent simulations convergence study: case 3. (a) horizontal and (b) vertical intensity cuts at E=7 keV for $me's$ ranging from 1 to 100k. (c) errors relative to the $me's$ =100k plots and (d) peak intensity.

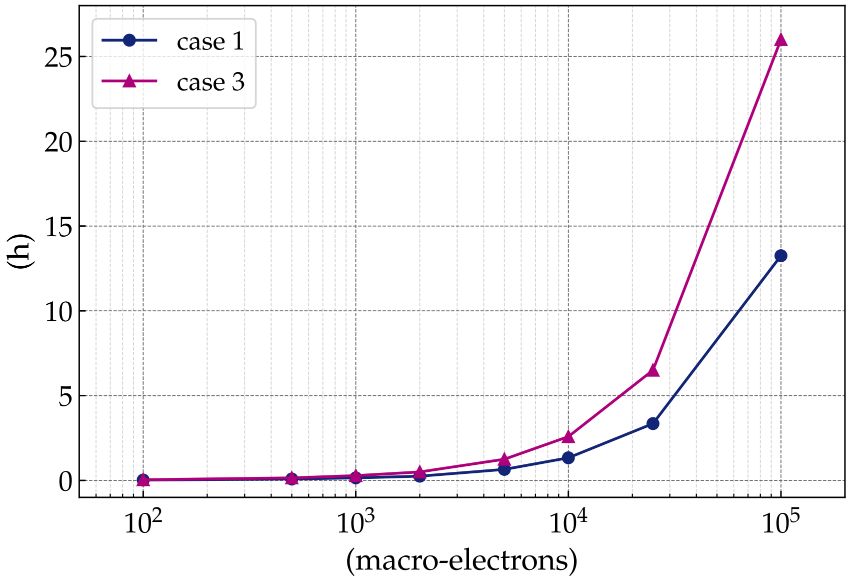

Fig. S3. Total elapsed time for partially-coherent simulations using a computer cluster with 28 processors for parallel calculations as a function of the number of $me's$.

References

Chubar, O., (2021). SRW GitHub repository (commit #38) in *https://www.github.com/ochubar/SRW.*

Chubar, O. & Celestre, R. (2019). *Opt. Express*, **27**(20), 28750–28759.

Cowley, J. M. & Moodie, A. F. (1957). *Acta Crystallographica*, **10**(10), 609.

He, A., Chubar, O., Rakitin, M., Samoylova, L., Fortmann-Grote, C., Yakubov, S. & Buzmakov, A. (2020). *Proc. SPIE 11493, Advances in Computational Methods for X-Ray Optics V*, pp. 78 – 87.

Kelly, D. P. (2014). *Journal of the Optical Society of America A*, **31**(4), 755.

Li, K. & Jacobsen, C. (2015). *J. Opt. Soc. Am. A*, **32**(11), 2074–2081.

Li, K., Wojcik, M. & Jacobsen, C. (2017). *Opt. Express*, **25**(3), 1831–1846.

Munro, P. R. T. (2019). *J. Opt. Soc. Am. A*, **36**(7), 1197–1208.

Paganin, D. M. (2006). *Coherent X-Ray Optics.* Oxford University Press.

Pirro, G. (2017). *Application of Scaled Wave Optics Propagator to Model Synchrotron Beamlines.* MSc thesis, Politecnico di Milano.

Sanchez del Rio, M., Wojdyla, A., Goldberg, K. A., Cutler, G. D., Cocco, D. & Padmore, H. A. (2020). *Journal of Synchrotron Radiation*, **27**(5), 1141–1152.

Schmidt, J. D. (2010). *Numerical Simulation of Optical Wave Propagation.* SPIE press.

iucr
